# Supplementary material for: Identifying and Addressing Basic Needs Insecurity Among Medical Students: A Curriculum for Trainees, Administrators, and Faculty
Source: MedEdPORTAL. 2022 Jan 10;18:11195. doi: 10.15766/mep_2374-8265.11195 (PMC8743318; doi:10.15766/mep_2374-8265.11195)
Supplement: Supplementary file 1 — Resource Guide.docxIn-Person Facilitator Guide.docxVirtual Facilitator Guide.docxPreworkshop Survey.docxBasic Needs Presentation.pptxCase 1.docxCase 2.docxCase 3.docxPostworkshop Survey.docx [file mep_2374-8265.11195-s001.zip › A. Resource Guide.docx]

**THIS DOCUMENT INCLUDES:**

1. **Basic Needs Survey**
   - This survey can be utilized to assess the basic needs insecurity the exists among the medical trainees at your institution. We recommend institutions implementing this workshop to perform this assessment prior to offering the session to quantify the problem in their student population. This is important for considering appropriate intervention strategies.
   - We suggest using Qualtrics, making the survey anonymous, and making the academic resource portion optional response for those students who may not have taken Step 1 or 2 yet and wish to complete the survey.
   - Medical students, faculty or staff/administration can create and distribute the survey and conduct the assessment.
2. **Programing Ideas + Resources List**
   - This is a list of ideas and national resources for your reference to help you initiate change at your institution.
   - Examples and links are provided for further reference.

**Basic Needs Survey**

1. **Demographics**
   1. With which of the following do you primarily identify?
      - Male
      - Female
      - Other
   2. With which of the following do you primarily identify?
      - White
      - Black or African American
      - Hispanic/Latinx
      - American Indian or Alaska Native
      - Asian
      - Native Hawaiian or Pacific Islander
      - Other
   3. What year medical student are you?
      - 1
      - 2
      - 3
      - 4
      - Other
2. **Food insecurity**
   - USDA (U.S Department of Agriculture)- 10 or 6 item scale
     - Most appropriate for students without children
     - USDA recommends using 10-item scale, but 6-item scale has been shown to give similar results
     - Can either assess last 30 days or 12 months
       - Recommended to survey both
     - Use items 1 or 2 below to assess food insecurity:
     - Reference: Hagedorn RL, Pampalone AL, Hood LB, Yura CA, Morrow DF, Olfert MD. Higher Education Food Insecurity Toolkit Development and Feedback. *J Nutr Educ Behav.* 2020;52(1):64-72.
3. USDA Food Security Survey Module: Six-Item Short Form
4. The food that I bought just did not last, and I didn’t have money to get more. Was that often, sometimes, or never true for you in the last 30 days (12 months)?
5. I couldn’t afford to eat balanced meals. Was that often, sometimes, or never true for you in the last 30 days (12 months)?
6. In the last 30 days (12 months), did you ever cut the size of your meals or skip meals because there wasn’t enough money for food?
7. [IF YES TO QUESTION 3, ASK] If using the 30 day version: In the last 30 days, how many days did this happen? If using the 12-month version: How often did this happen – almost every month, some months but not every month, or in only 1 or 2 months?

5. In the last 30 days (12 months) did you ever eat less than you felt you should because there wasn’t enough money for food?

6. In the last 30 days (12 months), were you ever hungry but didn’t eat because there wasn’t enough money for food?

2. USDA Food Security Module: Ten-Item Form

ADULT STAGE 1

1. “I worried whether my food would run out before I got money to buy more.” Was that often true, sometimes true, or never true for you in the last 30 days (12 months
2. “The food that I bought just didn’t last, and I didn’t have money to get more.” Was that often, sometimes, or never true for you in the last 30 days (12 months)?

3. “I couldn’t afford to eat balanced meals.” Was that often, sometimes, or never true for you in the last 30 days (12 months)?

IF THE RESPONDENT ANSWERS “OFTEN TRUE” OR “SOMETIMES TRUE” TO ANY OF THE THREE QUESTIONS IN ADULT STAGE 1, THEN PROCEED TO ADULT STAGE 2.

ADULT STAGE 2 (YES/NO QUESTIONS)

1. In the last 30 days (12 months, since last (name of current month)), did you ever cut the size of your meals or skip meals because there wasn’t enough money for food?
2. [IF YES TO QUESTION 4, ASK] If using the 30-day version: In the last 30 days, how many days did this happen? If using the 12-month version: How often did this happen – almost every month, some months but not every month, or in only 1 or 2 months?
3. In the last 30 days (12 months), did you ever eat less than you felt you should because there wasn’t enough money for food?
4. In the last 30 days (12 months), were you ever hungry but didn’t eat because there wasn’t enough money for food?
5. In the last 30 days (12 months), did you lose weight because there wasn’t enough money for food?

IF THE RESPONDENT ANSWERS “YES” TO ANY OF THE QUESTIONS IN ADULT STAGE 2, THEN PROCEED TO ADULT STAGE 3.

ADULT STAGE 3

1. In the last 30 days (12 months), did you ever not eat for a whole day because there wasn’t enough money for food?
2. [IF YES TO QUESTION 9, ASK] If using the 30-day version: In the last 30 days, how many days did this happen? If using the 12-month version: How often did this happen – almost every month, some months but not every month, or in only 1 or 2 months

1. **Housing insecurity**

- Difficult to assess- takes different forms depending on age and circumstances
  1. Crutchfield and Maguire’s (2017) instrument is based on definitions of homelessness developed by the U.S. Department of Housing and Urban Development and the U.S. Department of Education
- Crutchfield and Maguire’s Housing insecurity assessment

*choose to assess over 30-days or 12 months*

1. In the past 30 days (12 months), was there a rent or mortgage increase that made it difficult to pay?
2. In the past 30 days (12 months), did you not pay or underpay your rent or mortgage?
3. In the past 30 days (12 months), did you not pay the full amount of a gas, oil, or electricity bill?
4. In the past 30 days (12 months), have you moved two times or more?
5. In the past 30 days (12 months), did you move in with other people, even for a little while, because of financial problems?
6. In the past 30 days (12 months), did you “live with others beyond the expected capacity of the house or apartment”?

**4. Academic resources needs assessment**

- These are original questions designed by the authors of this resource.
- Step 1
  1. What resources did you wish to purchase?
     - Uworld Qbank
     - Sketchy Medical
     - First Aid
     - Pathoma
     - NBME Vouchers (for practice tests)
     - USMLE Rx Qbank
     - USMLE Rx Triple Play
     - Firecracker
     - Memorang
     - Osmosis
     - Kaplan Qbank
     - BoardVitals
     - Other - Write In
  2. What resources did you end up purchasing?
     - Uworld Qbank:
     - Sketchy Medical:
     - First Aid:
     - Pathoma
     - NBME Vouchers (for practice tests)
     - USMLE Rx Qbank:
     - USMLE Rx Triple Play
     - Firecracker:
     - Memorang:
     - Osmosis
     - Kaplan Qbank
     - BoardVitals
     - Other - Write In
  3. How much did you *expect* to spend on step 1 study materials (not including the cost of the exam?
     - Under $500
     - $500-$1000 (but not including $1000)
     - $1000-$2000 (but not including $2000)
     - $2000 and over
  4. What did you end up spending on Step 1 study materials (not including the cost of the exam)?
     - Under $500
     - $500-$1000 (but not including $1000)
     - $1000-$2000 (but not including $2000)
     - $2000 and over
- Step 2 CK
  1. What resources did you wish to purchase?
     - Uworld Qbank
     - Sketchy Medical
     - First Aid
     - Pathoma
     - NBME Vouchers (for practice tests)
     - USMLE Rx Qbank:
     - USMLE Rx Triple Play
     - Firecracker
     - Memorang
     - Osmosis
     - Kaplan Qbank
     - BoardVitals
     - Other - Write In (Required)
  2. What resources did you end up purchasing?
     - Uworld Qbank:
     - Sketchy Medical
     - First Aid:
     - Pathoma
     - NBME Vouchers (for practice tests)
     - USMLE Rx Qbank:
     - USMLE Rx Triple Play
     - Firecracker
     - Memorang
     - Osmosis
     - Kaplan Qbank
     - BoardVitals
     - Other - Write In (Required)
  3. How much did you *expect* to spend on Step 2 CK study materials (not including the cost of the exam?
     - Under $500
     - $500-$1000 (but not including $1000)
     - $1000-$2000 (but not including $2000)
     - $2000 and over
  4. What did you end up spending on Step 2 CK study materials (not including the cost of the exam)?
     - Under $500
     - $500-$1000 (but not including $1000)
     - $1000-$2000 (but not including $2000)
     - $2000 and over

**5. Transportation needs assessment**

1. Ideas taken from: Gould-Werth, Alix, Jamie Griffin, and Alexandra K. Murphy. 2018. “Developing a New Measure of Transportation Insecurity: An Exploratory Factor Analysis.” Survey Practice 11 (2). <https://doi.org/10.29115/SP-2018-0024>.
2. Transportation needs assessment:
3. Have you ever missed a class or important commitment due to an issue with transportation?
4. In the past 12 months, have you had to choose between transportation costs and paying for another necessity?
5. In the past 12 months, has there been a time when it was difficult to go somewhere due to the cost of public transportation?
6. In the past 12 months, has there been a time when you had difficulty getting where you needed to go because of a lack of public transportation routes?
7. Have you experienced or do you anticipate experiencing a time when your transportation situation limited the opportunities you were able to take advantage of?

**Programming Ideas and Resources**

**Addressing Basic Needs Insecurity**

- Ideas for faculty/staff/administrators
  - Identifying at risk students early on and setting them up with a mentor
    - Using medical school applications to look for – first-generation students, students who received the Federal Pell Grant

**Addressing Food Insecurity**

- Ideas for students and faculty/staff/administrators
  - Create a “take what you need” resource pantry on campus
    - Example) UCF campus pantry for medical and graduate students
      - <https://www.ucf.edu/news/med-school-adds-knights-pantry-aid-students/>
    - Example) UMass Food Pantry
      - [Max Baker Resource Center for students facing food insecurity dedicated at UMMS (umassmed.edu)](https://www.umassmed.edu/news/news-archives/2018/11/max-baker-resource-center-for-students-facing-food-insecurity-dedicated-at-umms/)
  - Form local partnerships
    - Talk with local food banks, grocery stores, religious organizations, salvation army
    - Implement student discounts or donations
  - Create a campus garden
    - Grow fresh fruits and vegetables that students can utilize
    - Example) University of Michigan Medical School has successfully created a Medical Campus Garden
      - https://medicine.umich.edu/medschool/student-groups/medical-campus-garden
  - Skills workshop (Run by faculty/administrators/staff)
    - Implement a lifestyle skill workshop into freshman orientation.
    - Discuss budgeting and cooking to ensure students are making the most of their budget and groceries.
- National Resources
  - College and University Food Bank Alliance (CUFBA)
    - Digital community to help support, train and provide resources for students wanting to start a food bank
    - Link: https://characterclearinghouse.fsu.edu/article/college-university-food-bank-alliance
  - Apply for SNAP (Supplemental Nutrition Program)
  - Federal Student loans (refer to pg. 10 for more info)

**Addressing Housing Insecurity**

- Ideas for faculty/staff/administrators
  - Call rooms
  - Library nap areas
    - Examples) Michigan, Berkley and Wesleyan designate napping zones
      - <https://www.libraryjournal.com/?detailStory=u-michigan-libraries-open-nap-stations>
      - <https://www.lib.berkeley.edu/libraries/moffitt-library/rest-zone>
      - <https://newsletter.blogs.wesleyan.edu/2012/10/22/energypods/>
- National Resources
  - Home Energy Assistance Programs (HEAP)
  - GA/RA for university college house
  - Churches and Homeless Shelters
  - Federally subsidized housing- Section 8 & Section 42
  - Federal Student loans (refer to pg. 10 for more info)

**Addressing Academic Resource Insecurity**

- Ideas for faculty/staff/administrators
  - School funded/discounted resources
    - Example) <https://guides.library.nymc.edu/c.php?g=935825&p=6744777>
      - NYMC makes Pathoma and First Aid e-books available to second year medical students
    - Example) <https://medicine.umich.edu/medschool/education/md-program/financial-aid/extra-help-current-students/supplemental-funds>
      - University of Michigan allows students to apply for reimbursements for administratively mandated USMLE Step 1 and Step 2 review courses (ex: Stanley Kaplan).
  - Library- Borrow Direct, EZ Borrow, Interlibrary Loan
  - Advocating for waivers at the state level
- Ideas for both students and faculty/staff/administrators
  - Student fundraising events
  - Book sale/exchange
  - Hold a financial workshop
    - Many students are unaware of the cost of USMLE tests and materials which results in the lack of proper budgeting
    - Use data from academic resources section of the Basic Needs Survey above
- National Resources
  - Federal Student loans (refer to pg. 10 for more info)

**Addressing Transportation Insecurity**

- Ideas for faculty/staff/administrators
  - School provided public transit passes (included in tuition)
- Ideas for both students and faculty/staff/administrators
  - School organized platform to find a carpool
    - Example) Carpooling program at Emory University
      - <http://transportation.emory.edu/commute/carpool/index.html>
- National Resources
  - Federal Student loan (refer to pg. 10 for more info)

**Student Loans**

- This section is meant for both students to refer to and faculty/staff/administrators to be familiar with when students come to them with questions regarding loans.
- Faculty/administrations can present these resources to student but should also refer students to the financial aid office at their institutions where they can receive formal counseling.

**Types of loans**

- Federal Direct Unsubsidized Loans
  - Non-credit based loan based on a student’s cost of attendance.
  - Interest accrues while in school.
  - Begin paying back 6 months after graduation or if less than half-time student.
- HRSA Primary Care Loan
  - For students who agree to complete residency in primary care and must practice primary care for 10 year or until the loan is paid in full.
  - Must meet certain income guidelines.
  - Limited funds.
- Federal Direct Grad PLUS Loans
  - A loan that requires a credit check.
  - This is a good loan to use after if students still require more aid after unsubsidized loans.
  - Interest in higher than federal unsubsidized loans.
  - Interest accrues while in school.
- More information on most common types of loans
  - https://www.aamc.org/professional-development/affinity-groups/gea/first-generation-students#financial

**Additional resources for students**

- AAMC financial page
  - <https://students-residents.aamc.org/financial-aid>
- AAMC budget worksheet for students
  - https://students-residents.aamc.org/media/5131/download

**Residency relocation**

- It takes some time for residents to be put on the payroll of their new institution, which can lead to several weeks between graduation and their first paycheck without any loan support. Below are resources for addressing this issue.
- Resources/Ideas
  - See if your institution has loans specifically for residency relocation
    - Example at Bostin University) https://www.bumc.bu.edu/osfs/resources/types-of-aid/loans/creditbasedloans/rrloans/
  - Talk to alumni networking services
    - May help new residents find temporary housing during this transition
  - Lease guaranty programs
    - Refer here for more information - https://www.bmc.org/medical-professionals/graduate-medical-education-gme/house-staff/intern-resident-and-fellow-benefits#Orientation_Pay
