# Supplementary material for: Identifying and Addressing Basic Needs Insecurity Among Medical Students: A Curriculum for Trainees, Administrators, and Faculty
Source: MedEdPORTAL. 2022 Jan 10;18:11195. doi: 10.15766/mep_2374-8265.11195 (PMC8743318; doi:10.15766/mep_2374-8265.11195)
Supplement: Supplementary file 1 — Resource Guide.docxIn-Person Facilitator Guide.docxVirtual Facilitator Guide.docxPreworkshop Survey.docxBasic Needs Presentation.pptxCase 1.docxCase 2.docxCase 3.docxPostworkshop Survey.docx [file mep_2374-8265.11195-s001.zip › C. Virtual Facilitator Guide.docx]

*Before the session:*

- Create a Zoom link
- Create pre- and post-workshop surveys to evaluate the knowledge of the learners.
  - We recommend using Qualtrics.
- Insert required information into the PowerPoint presentation.
  - Facilitator names, survey QR codes/links.
- Assign positions:
  - **Facilitator 1 & 2**
    - Facilitator 1:
      - PowerPoint Presentation (Slides 1-21)
      - In breakout room: Case 2 (Harry)
    - Facilitator 2:
      - Case 1 (Gina) as a large group (Slides 23-24)
      - In breakout room: Case 3 (Joe)
      - PowerPoint Presentation (Slides 30-36)

- - - Say: “next slide” when finished
      - The technical moderator should oversee the changing the PowerPoint slides.
  - **Technical moderator**
    - Role:
      - Change the slides for the facilitators.
      - In charge of watching the time & signaling when to move on.
        - If facilitator is running over, send a personal chat message to move on.
      - Managing the breakout room feature
        - Utilize Zoom instructions on the details of how to generate 2 randomly generated breakout rooms.
- Print/send out materials:
  - Facilitator Guide
    - 1 for each facilitator + technical moderator
  - Resource Guide
    - Send to participants via email just prior to the workshop.
    - Print 1 for each facilitator for reference during the workshop.

*Just prior to the session:*

1. All facilitators log onto Zoom link 30 minutes prior to schedule start time.
2. Technical moderator - Set the zoom settings:
   1. Make all facilitators co-hosts.
   2. Allow participants to chat with anyone.
   3. Mute all participants (default), allow participants to unmute themselves.
   4. Open the PowerPoint Presentation.

**Prior to Official Start**

- Facilitator 1:
  - Ask participants to complete **pre-evaluation** **survey** as they join via
    - 1) QR code on the slide OR
    - 2) Send pre-evaluation link in chat
  - Ask participants to have the **Resource Guide available** for reference

**Slide 1: Introduction (5 min)**

- Introduction:
  - Facilitators + Technical moderator introduce themselves
  - Thank everyone for coming to our workshop, “It’s Time to Talk About Basic Needs Insecurity Among Medical Trainees”
- Facilitator 1:
  - To help us connect in this virtual environment, **ask participants** to please enter their **first name, institution, and reason for joining the workshop** into the chat
    - **Read a few responses** and welcome everyone
  - Ask participants if they have not already done so to complete **pre-evaluation** **survey** via
    - 1) QR code on the slide OR
    - 2) Send pre-evaluation link in chat and ask participants to complete it.
  - When done with pre-survey, ask participants **to pull up Resource Guide**
    - May want to refer to it later during the case discussions.
- (Optional) Facilitator 2:
  - Check the number of participants who completed the pre-evaluation before moving forward.
    - Ex) If there are 15 participants total: “We have 10 completed surveys, so we are still waiting for about 5 more people to finish up”.

**Slides 2 + 3: Learning Objectives & Agenda** **(1 min total)**

- Facilitator 1:
  - Read learning objectives
  - Read Agenda – **Bold terms**

**Slides 4: What is the definition? (1 min)**

- Facilitator 1:
  - **Ask participants** to use the **chat box** to answer the question
  - **Read responses** as they come in

**Slides 5: Basic Needs Definition (30 sec)**

- Facilitator 1:
  - Read the 4 basic needs of medical students
    - Food
    - Shelter
    - Transportation
    - Academic Resources

**Slides 6: Statistics (30 sec)**

- Facilitator 1:
  - Explain these are some **background statistics** from a national report from a **survey administered in 2018 to two- and four- year institutions across the US in which 86,000 students participated**
  - We are looking at undergraduate stats because this is the largest study to our knowledge of basic needs insecurity among higher education students.

**Slides 7: Food Insecurity Definition (30 sec)**

- Facilitator 1:
  - Read the key identifiers of food insecurity
    - Limited or uncertain access to nutritious food

**Slides 8: How Prevalent is this? (30 sec)**

- Facilitator 1:
  - **Ask participants** to use the **chat box** to answer the question
  - **Read responses** as they come in

**Slides 9: Food Insecurity Statistics (30 sec)**

- Facilitator 1:
  - Read **statistic**
    - **28.5 %** of surveyed **professional students** (medical, dental, etc.) met the criteria for food insecurity.
    - **1 in 5** of these students reported that their food insecurity negatively impacted their academic performance.

**Slides 10: Food Insecurity Statistics (30 sec)**

- Facilitator 1:
  - Point out that **the rate of food insecurity varies by race and ethnicity**
    - Highlight two statistics.
  - Point out that **the rate of food insecurity also varies by gender identity**
    - Highlight two statistics.

**Slides 11: Food Insecurity Strategies (30 sec)**

- Facilitator 1:
  - Read 3 example strategies to support food insecure students
    - Create a campus garden
    - Create a resource pantry
    - Apply for SNAP
    - Student loans / Scholarship applications– can ask the financial aid office to help (Federal unsubsidized loans, Federal GRAD PLUS loans, etc.)

**Slides 12: Housing Insecurity Definition (30 sec)**

- Facilitator 1:
  - Read the key identifiers of housing insecurity
    - Inability to pay rent or utilities
    - Unstable living situation

**Slides 13: How Prevalent and What Are Risk Factors? (30 sec)**

- Facilitator 1:
  - **Ask participants** to use the **chat box** to answer the questions
  - **Read responses** as they come in

**Slides 14: Housing Insecurity Statistics (30 sec)**

- Facilitator 1:
  - Read **statistic** **from the previously mentioned survey**
    - 55% of surveyed college students experienced housing insecurity in the previous year
  - Read **most predictive factor**
    - Parent education level
    - **64%** of students whose parents did not graduate high school experience housing insecurity
  - Point out that like food insecurity, **the rate of housing insecurity also varies by race and ethnicity**

**Slides 15: Housing Insecurity Strategies (30 sec)**

- Facilitator 1:
  - Read 3 example strategies to support housing insecure students
    - Create a library nap area
    - Apply for HEAP – to help with utilities
    - Offer free housing through graduate assistant or resident assistant programs
      - If the school has an associated undergraduate institution
    - Student loans / Scholarship applications– can ask the financial aid office to help (Federal unsubsidized loans, Federal GRAD PLUS loans, etc.)

**Slides 16: Transportation Insecurity Definition (30 sec)**

- Facilitator 1:
  - Read the key identifiers of transportation insecurity
    - Inability to travel in a **safe** and **timely** manner due to the lack of resources

**Slides 17: Transportation Insecurity Strategies (30 sec)**

- Facilitator 1:
  - Read 3 example strategies to support transportation insecure students
    - Organize a carpooling program
    - Provide public transit passes
    - Provide free parking passes
    - Provide gas stipends for rotations that are far away

**Slides 18: Academic Insecurity Definition (30 sec)**

- Facilitator 1:
  - Read the key identifiers of academic resource insecurity
    - Difficulty or inability to afford required or recommended resources to be adequately prepared

**Slides 19: How Prevalent is Academic Resource Insecurity? (30 sec)**

- Facilitator 1:
  - **Ask participants** to use the **chat box** to answer the questions
  - **Read responses** as they come in

**Slides 20: Academic Insecurity Statistics (30 sec)**

- Facilitator 1:
  - Read statistics
    - **61%** of students thought they’d spend **under $500** studying for Step 1
    - **Over 50%** of students spent over $500
      - This discrepancy is important for budgeting purposes!

**Slides 21: Academic Insecurity Strategies (30 sec)**

- Facilitator 1:
  - Read 3 example strategies to support academic resource insecure students
    - School funded/discounted study materials
    - School library resources
    - Organize a book sale or exchange

**Slides 22: Case Studies (30 sec)**

- Facilitator 2:
  - Explain **we will now discuss three case studies,** the first as a large group and the second two in breakout rooms
  - We will **read through the case,** then **discuss questions** surrounding the case

**Slides 23: Case 1 – Gina (1 minute)**

- Facilitator 2:
  - Read the case

**Slides 24: Case 1 Discussion – Gina (9 minutes)**

- Facilitator 2:
  - Read the questions and ask participants to answer in the chat
  - **Read background information** with participants as relevant answers come up

**Discussion Questions:**

**Questions 1:** What basic needs insecurities is Gina dealing with and what are the consequences of these insecurities?

Probing:

Encourage learners to brainstorm the effects of Gina’s food insecurity on her physical and mental health, sleep quality, academic performance, career aspirations, burnout, scholarly activity, etc. Reflect the effect on patient care.

Background information:

- Food insecurity: The limited or uncertain availability of nutritionally adequate and safe food, or the ability to acquire such food in a socially acceptable manner.
- Academic resource insecurity: The difficulty or inability to afford required or recommended resources to be properly prepared for academics.
- Sleep and stress: A multi-institution study of college students in the US found food insecure students are 4x more likely to have high perceived stress and 2x more likely to have poor sleep quality^1^.
- Academics: When controlling for sociodemographic characteristics, food-insecure students had almost twice the risk of GPA <3.00 compared to their food-secure counterparts^1^.

**Question 2:** What demographic information puts Gina at risk for these insecurities?

Discussion: Understanding the risk factors for food insecurity can help identify students in need of resources and support. Encourage learners to **consider first-generation status, minority status, single-parent households**, etc.

Background information:

- Risk factors: Government Accountability Office (GAO) analyzed 31 studies on food insecurity among college students and **found low-income was the most common risk factor**, among these students most are associated with either being a first-generation student or a single parent^2^.

**Question 3:** What community assets/resources are available for her in your community? What barriers might exist to accessing those resources and services?

Discussion: Ponder resources in the community (food pantries, local student discounted restaurants). Consider ways to eliminate the stigma of utilizing food pantries / accepting aid from available resources.

Background information:

- On campus food pantries: Most food-insecure college students (77.8%) did not utilize the pantry for food acquisition and most students preferred a central location for the pantry while 1/3 preferred a hidden location^1^.
- Federal benefit programs: Supplemental Nutrition Assistance Program (SNAP) is underutilized and one of the contributing factors is the ambiguity of who is eligible^2^.
- Loans are always important to discuss – refer to pg. 10 Resource Guide for more background information.

**Question 4**: What questions can we ask Gina to assess for the basic needs insecurities she may be experiencing? *(remember a Resource Guide is available as a resource)*

**Transition:** **(2 minute)**

- Facilitator 2:
  - “We will now transition into two breakout rooms. Each breakout room will discuss one case each. The cases we will be using have been sent in the chat. Please assign someone in your group to share what your group discussed when we come back together.”
- Technical Moderator:
  - **Send** the file with **cases in the chat**
  - **Open** 2 breakout rooms
  - **Insure** there is **1 facilitator in each room**
  - **Set a timer for 8 minutes**

**Facilitator 1**

**Case 2 – Harry (1 minute)**

- Read the case

**Case 2 Discussion – Harry (9 minutes)**

- Read the questions and ask participants to unmute themselves to answer or put it in the chat
- **Read background information** with participants as relevant answers come up

**Discussion Questions:**

**Questions 1:** What basic needs insecurities is Harry dealing with and what are the consequences of these insecurities?

Probing:

Encourage learners to brainstorm the effects of Harry’s housing insecurity on his physical and mental health, sleep quality, academic performance, career aspirations, burnout, scholarly activity, etc. Reflect the effect on patient care.

Background information:

- Housing insecurity and psychosocial effects:
  - A study investigated the effects of housing and parental insecurity on youths and found that “the additive effects of instability in housing and parental care are strongly associated with diminished healthy psychosocial functioning^3^.
- Housing Insecurity and academic performance:
  - The same study shows that housing insecurity leads to worse academic performance and achievement^3^.
  - “Our findings expand this understanding regarding homeless populations by showing significant differences within dual instabilities, using GPA as an indicator of academic success. Youth in single and both instability groups fare significantly worse on all available measures of school achievement. They are less likely to be engaged and committed to their school experiences. They report less involvement with their school communities, and they receive less encouragement and fewer opportunities to use their school environments as a foundation for prosocial development”^3^.
- Housing Insecurity and Food Insecurity:
  - A study on food insecurity among college students found that college students who were dealing with housing instability, were at an increased risk of being food insecure: “housing instability significantly (AOR = 8.00, 95%CI=3.57–17.93, P-value<0.0001) increased the odds of being food insecure^4^.

**Question 2:** How could we have identified students experiencing these insecurities before the circumstances progressed to Harry’s level?

Probing:

Understanding the risk factors for housing insecurity can help identify students in need of resources and support. Encourage learners to consider first-generation status, minority status, single-parent households, etc.

Perhaps surveying students – using the basic needs insecurity survey that we will discuss later in the presentation.

**Question 3:** What community or school assets/resources may be available for him on your campus? *Remember a Resource Guide is available to help as you identify potential resources.*

Probing

Ponder resources in the community (Home Energy Assistance Programs (HEAP), GA/RA for university college house, Churches and Homeless Shelters, Federally subsidized housing- Section 8 & Section 42). Consider ways to eliminate the stigma of utilizing these resources/ accepting aid from available resources.

Background information:

- “Without positive school climates and supportive school staff, services for youth with housing and related instabilities may not reach them until they are already in deeply harmful circumstances. School staff are gatekeepers to supports for youth experiencing instability.^3^
- Loans are always important to discuss – refer to pg. 10 Resource Guide for more background information.

**Facilitator 2**

**Case 3 – Joe (1 minute)**

- Read the case

**Case 3 Discussion – Joe (9 minutes)**

- Read the questions and ask participants to unmute themselves to answer or put it in the chat
- **Read background information** with participants as relevant answers come up

**Discussion questions:**

**Questions 1:** What basic needs insecurities is Joe dealing with and what are the consequences of these insecurities?

Probing:

Explore the additional basic needs insecurities that arise during the third year for medial trainees. How can we best prepare, educate, and support students when transportation becomes yet another barrier some students have navigate in a timely manner?

Background:

Transportation insecurity: Inability to regularly move from place to place in a safe and timely manner because one lacks the resources one needs for transportation

- **Question 2:** How far in advance should students be told about extra transportation costs to allow adequate time to plan/budget?

Probing:

Encourage learners to contemplate the impact of timing in terms of when Joe’s institutions gives him his clerkship locations and inform him of these extra costs.

- **Question 3:** What community or school assets/resources may be available for him?

Probing:

Encourage learners to contemplate what Joe’s institution can provide for students, how public transportation may be utilized, etc.

Background:

- Providing students with public transportation memberships.
- Established carpooling opportunities for students.
- Gas vouchers or reimbursement.
- Loans are always important to discuss – refer to pg. 10 Resource Guide for more background information.

**Regroup (1 min)**

- Technical Moderator:
  - **Close** breakout rooms after **8 minutes**

**Slide 30: Sharing (6 minutes – 3 minutes/group)**

- Facilitator 2:
  - “We would now like to hear from each group, if one member from each group would like to please unmute themselves and share what you discussed in your small group”
  - “Let’s start with Case Study 2 about Harry”
  - **Facilitate discussion** around that case and **invite others to join in** as time allows (3 minutes)
  - “Thank you for that. Let’s move on to Case Study 3 about Joe, would a member of that group please unmute themselves and share what you discussed.”
  - **Facilitate discussion** around that case and **invite others to join** in as time allows (3 minutes)

**Slide 31: Goals of the Workshop (30 sec)**

- Facilitator 2:
  - To show that basic needs insecurity **exists** among graduate and professional students
  - Key points
    - Basic needs insecurity is very prevalent and under documented in graduate and professional students
    - There are risk factors for these basic needs insecurities
    - There are programs to support students which they should be made aware of

**Slides 32: Goals of the Workshop (30 sec)**

- Facilitator 2:
  - To motivate other to explore, assess, and address basic needs insecurity on their campus
  - To provide tools and resources to support efforts to assess and address basic needs insecurity.
    - Examples can be found on the Resource Guide

**Slides 33: Assessment Example (30 sec)**

- Facilitator 2:
  - This is an example of one can assess food insecurity using the USDA Food Security Survey Module which can be found on the Resource Guide.
  - This survey, and others regarding housing, transportation, and academic resource insecurity can all be found in the basic needs survey that our team has put together on the Resource Guide.

**Slides 34: Post-Workshop Evaluation (5 minutes)**

- Facilitator 2:
  - “Please complete the post-workshop evaluation by scanning the QR code with your phone or clicking the link in the chat.”
- Technical Moderator:
  - Paste post-workshop evaluation link into the chat

**Slides 35: Questions (5 minutes)**

**Slides 36: Thank you (30 seconds)**

References

1. El Zein A, Shelnutt KP, Colby S, et al. Prevalence and correlates of food insecurity among U.S. college students: a multi-institutional study. *BMC Public Health.* 2019;19(1):660.
2. https://www.gao.gov/products/GAO-19-95. Better Information Could Help Eligible College Students Access Federal Food Assistance Benefits. https://www.gao.gov/products/GAO-19-95. Published December 21, 2018. Accessed February 9, 2021.
3. Crumé HJ, Nurius PS, Fleming CM. Cumulative Adversity Profiles Among Youth Experiencing Housing and Parental Care Instability. *Child Youth Serv Rev.* 2019;100:129-135.
4. Payne-Sturges DC, Tjaden A, Caldeira KM, Vincent KB, Arria AM. Student Hunger on Campus: Food Insecurity Among College Students and Implications for Academic Institutions. *Am J Health Promot.* 2018;32(2):349-354.
