# Supplementary material for: Identifying and Addressing Basic Needs Insecurity Among Medical Students: A Curriculum for Trainees, Administrators, and Faculty
Source: MedEdPORTAL. 2022 Jan 10;18:11195. doi: 10.15766/mep_2374-8265.11195 (PMC8743318; doi:10.15766/mep_2374-8265.11195)
Supplement: Supplementary file 1 — Resource Guide.docxIn-Person Facilitator Guide.docxVirtual Facilitator Guide.docxPreworkshop Survey.docxBasic Needs Presentation.pptxCase 1.docxCase 2.docxCase 3.docxPostworkshop Survey.docx [file mep_2374-8265.11195-s001.zip › D. Preworkshop Survey.docx]

Preworkshop Survey

Q1 What is your current gender identity? Select all that apply.

- Male
- Female
- Trans male/Trans man
- Trans female/ Trans woman
- Genderqueer / Gender non-conforming
- Different Identity

Q2 What sex were you assigned at birth?

- Male
- Female

Q3 Please check all that apply:

- Latinx/Hispanic
- Black/African American
- White
- Asian
- Native American/Alaskan or Pacific Islander
- Other: ________________________________________________

Q4 Which of the following best describes you?

- Undergraduate Student
- Medical Student
- Medical Resident or Fellow
- Staff member/Administrator
- Academic faculty/Clinician (MD, DO, NP, PA, Other)
- Other: Please specify ________________________________________________

Q5 Do you consider yourself a first-generation college graduate (i.e. having no parent with a 4 year college degree)

- Yes
- No

Q6 Did you receive the Federal Pell Grant in undergraduate education?

- Yes
- No

Q7 In what state is your academic institution located?

________________________________________________________________

Q8 Which is a basic need of medical students?

- **Academic resources to ensure they succeed.**
- A car so they can get to clinical rotations.
- A mentor they trust for career advice.
- A free gym membership to maintain their physical and mental health.

Q9 What percent of professional students are food insecure?

- 5%
- **28.5%**
- 45%
- 75.9%

Q10 What percent of college students experience housing insecurity?

- 5%
- 15%
- **55%**
- 70%

Q11 Which student characteristic is most predictive of their likelihood for experiencing housing insecurity?

- Race/Ethnicity - American Indian/Alaskan Native students
- **Parental Education - Students whose parents do not have a high school diploma**
- Older Students - Students aged 26-30
- Sexual Orientation - Gay and lesbian students

Q12 Which medical student is available for Supplemental Nutrition Assistance Program (SNAP) benefits?

- No medical student, it is only for undergraduate students
- **A medical student with work study job**
- A medical student whose parents make <$100,000 annually
- A medical student who is in the top 15% of their class
