# Supplementary material for: Identifying and Addressing Basic Needs Insecurity Among Medical Students: A Curriculum for Trainees, Administrators, and Faculty
Source: MedEdPORTAL. 2022 Jan 10;18:11195. doi: 10.15766/mep_2374-8265.11195 (PMC8743318; doi:10.15766/mep_2374-8265.11195)
Supplement: Supplementary file 1 — Resource Guide.docxIn-Person Facilitator Guide.docxVirtual Facilitator Guide.docxPreworkshop Survey.docxBasic Needs Presentation.pptxCase 1.docxCase 2.docxCase 3.docxPostworkshop Survey.docx [file mep_2374-8265.11195-s001.zip › E. Basic Needs Presentation.pptx]

## Slide 1
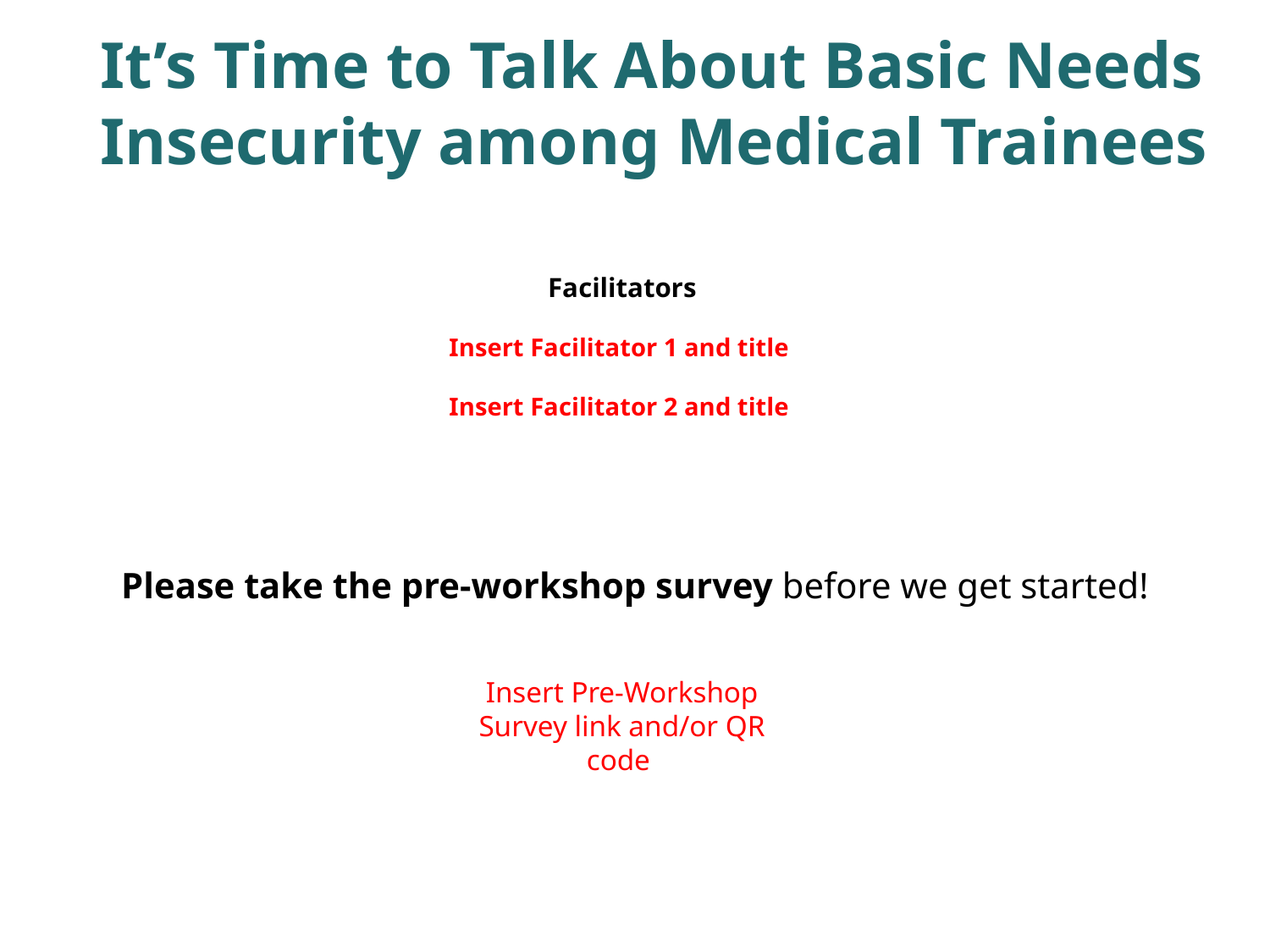

It’s Time to Talk About Basic Needs Insecurity among Medical Trainees
Facilitators
Insert Facilitator 1 and title
Insert Facilitator 2 and title
Please take the pre-workshop survey before we get started!
Insert Pre-Workshop Survey link and/or QR code

## Slide 2
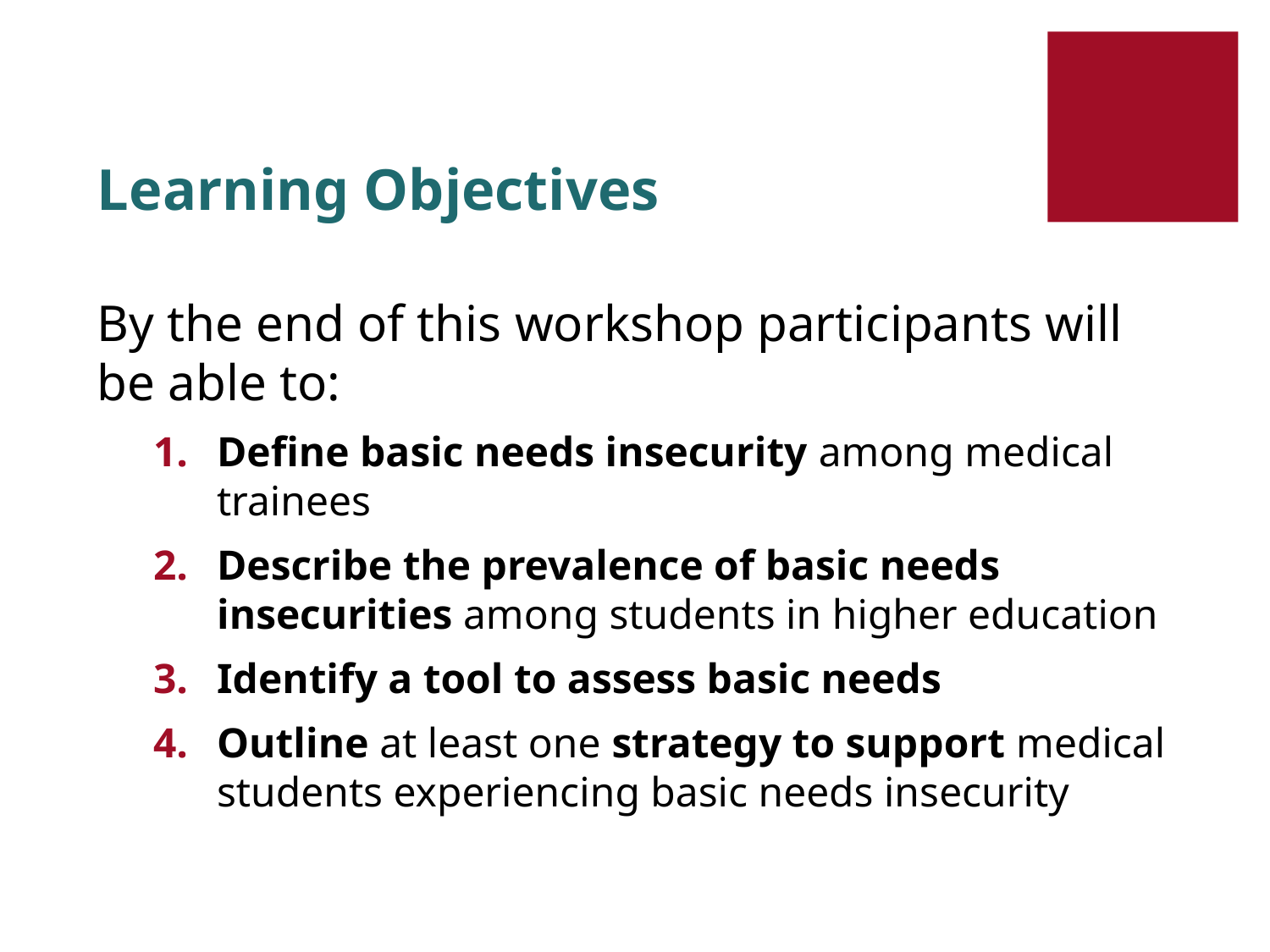

# Learning Objectives
By the end of this workshop participants will be able to:
Define basic needs insecurity among medical trainees
Describe the prevalence of basic needs insecurities among students in higher education
Identify a tool to assess basic needs
Outline at least one strategy to support medical students experiencing basic needs insecurity

## Slide 3
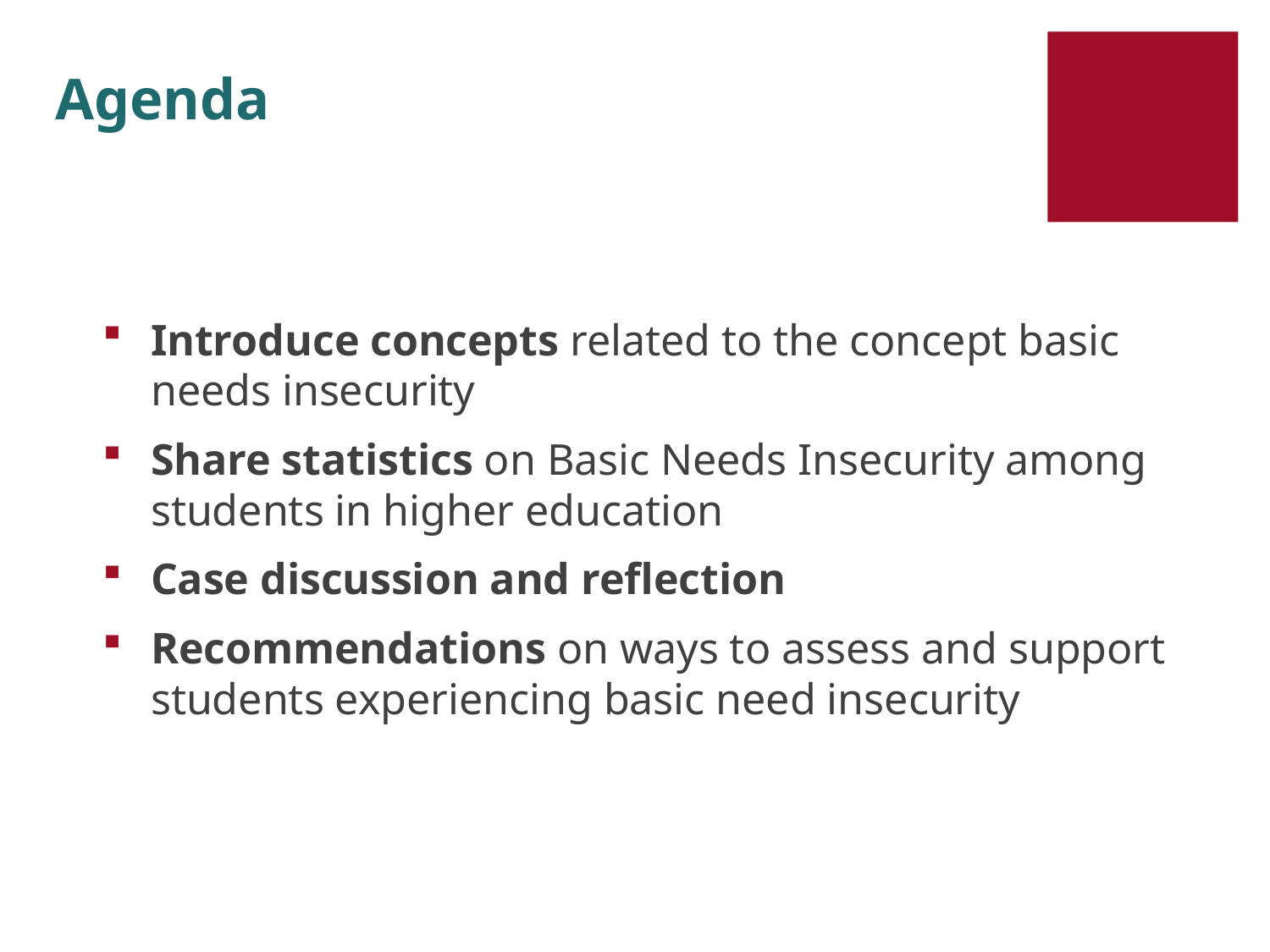

# Agenda
Introduce concepts related to the concept basic needs insecurity
Share statistics on Basic Needs Insecurity among students in higher education
Case discussion and reflection
Recommendations on ways to assess and support students experiencing basic need insecurity

## Slide 4
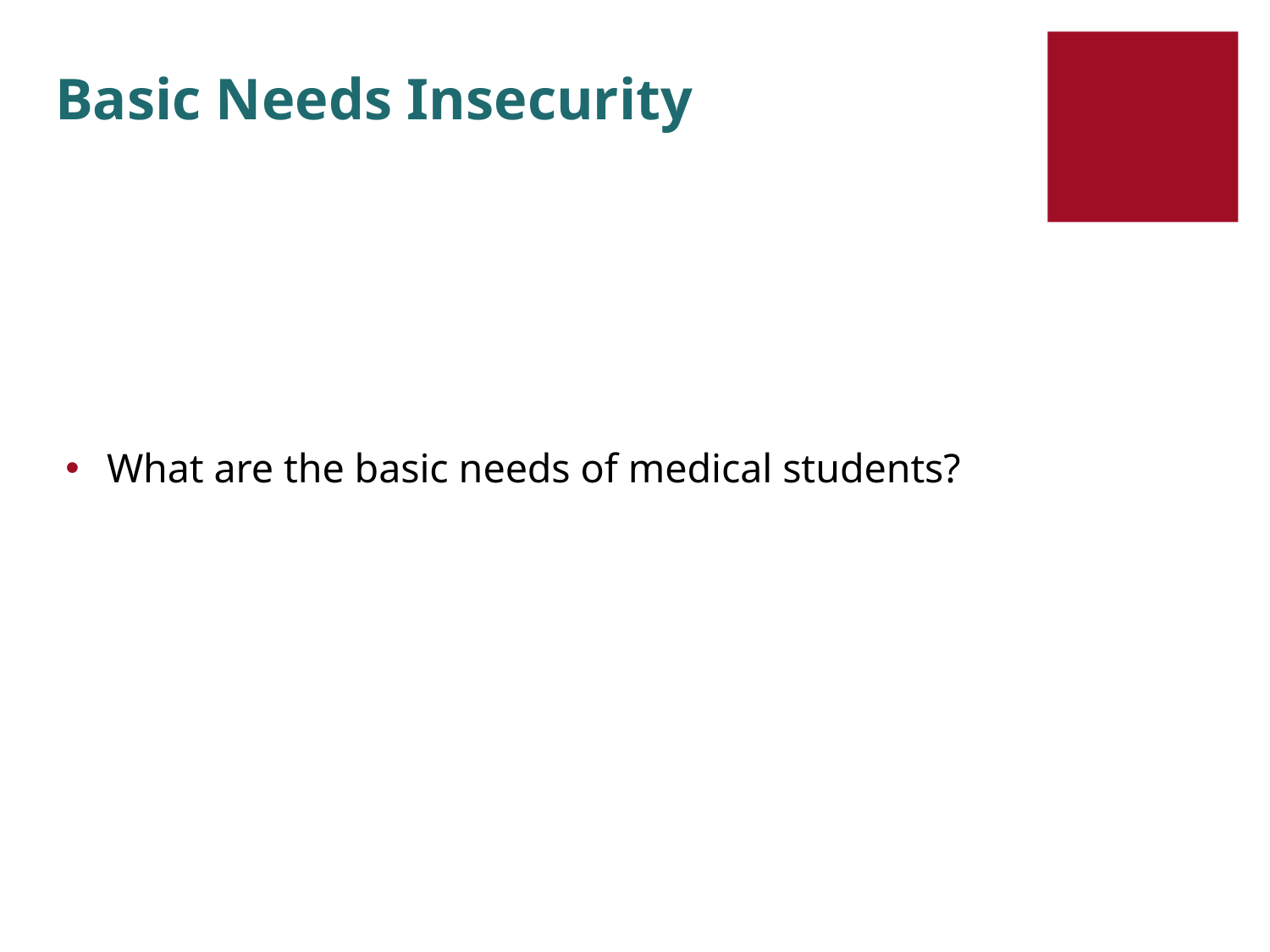

# Basic Needs Insecurity
What are the basic needs of medical students?

## Slide 5
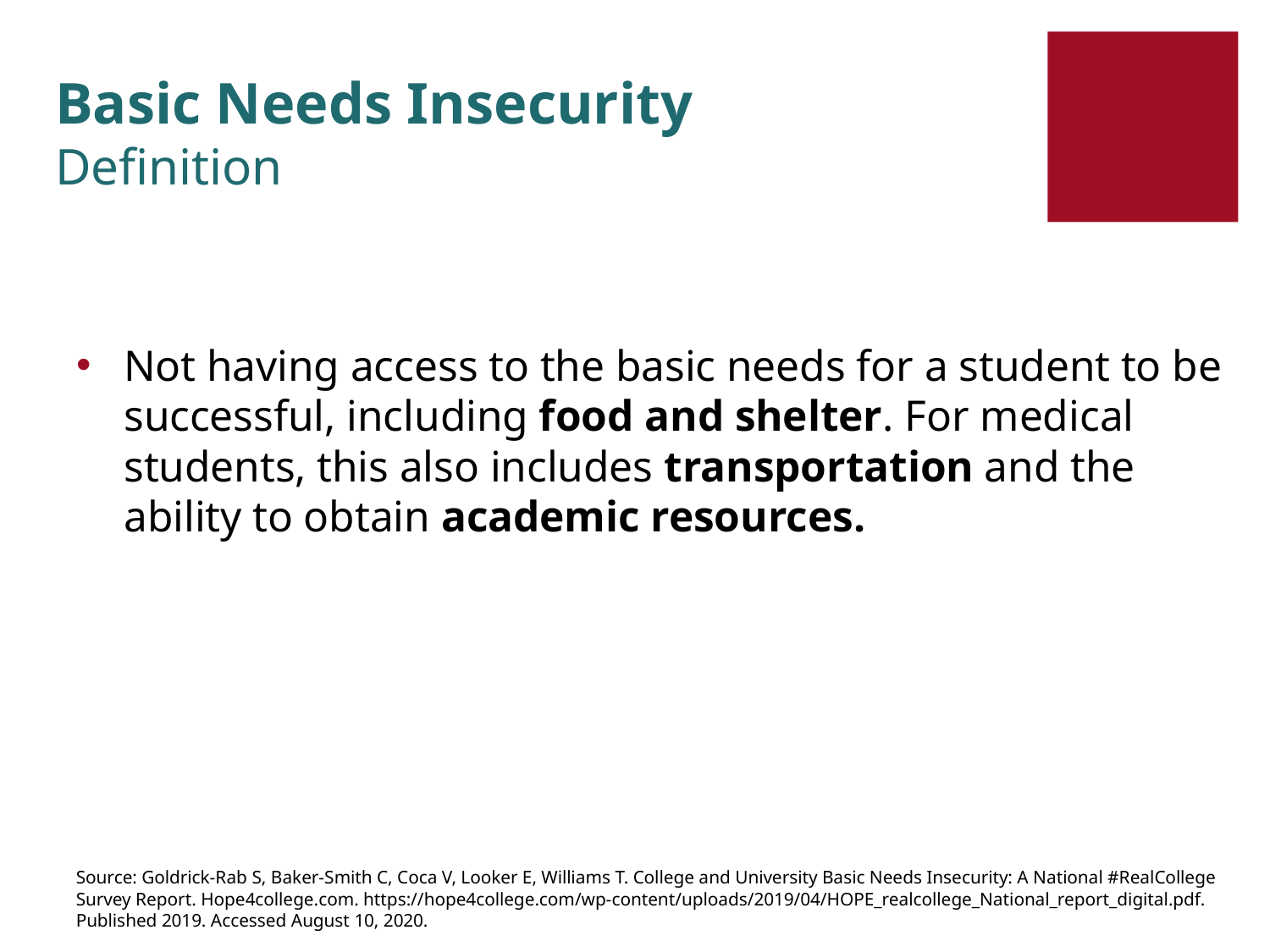

# Basic Needs Insecurity Definition
Not having access to the basic needs for a student to be successful, including food and shelter. For medical students, this also includes transportation and the ability to obtain academic resources.
Source: Goldrick-Rab S, Baker-Smith C, Coca V, Looker E, Williams T. College and University Basic Needs Insecurity: A National #RealCollege Survey Report. Hope4college.com. https://hope4college.com/wp-content/uploads/2019/04/HOPE_realcollege_National_report_digital.pdf. Published 2019. Accessed August 10, 2020.

## Slide 6
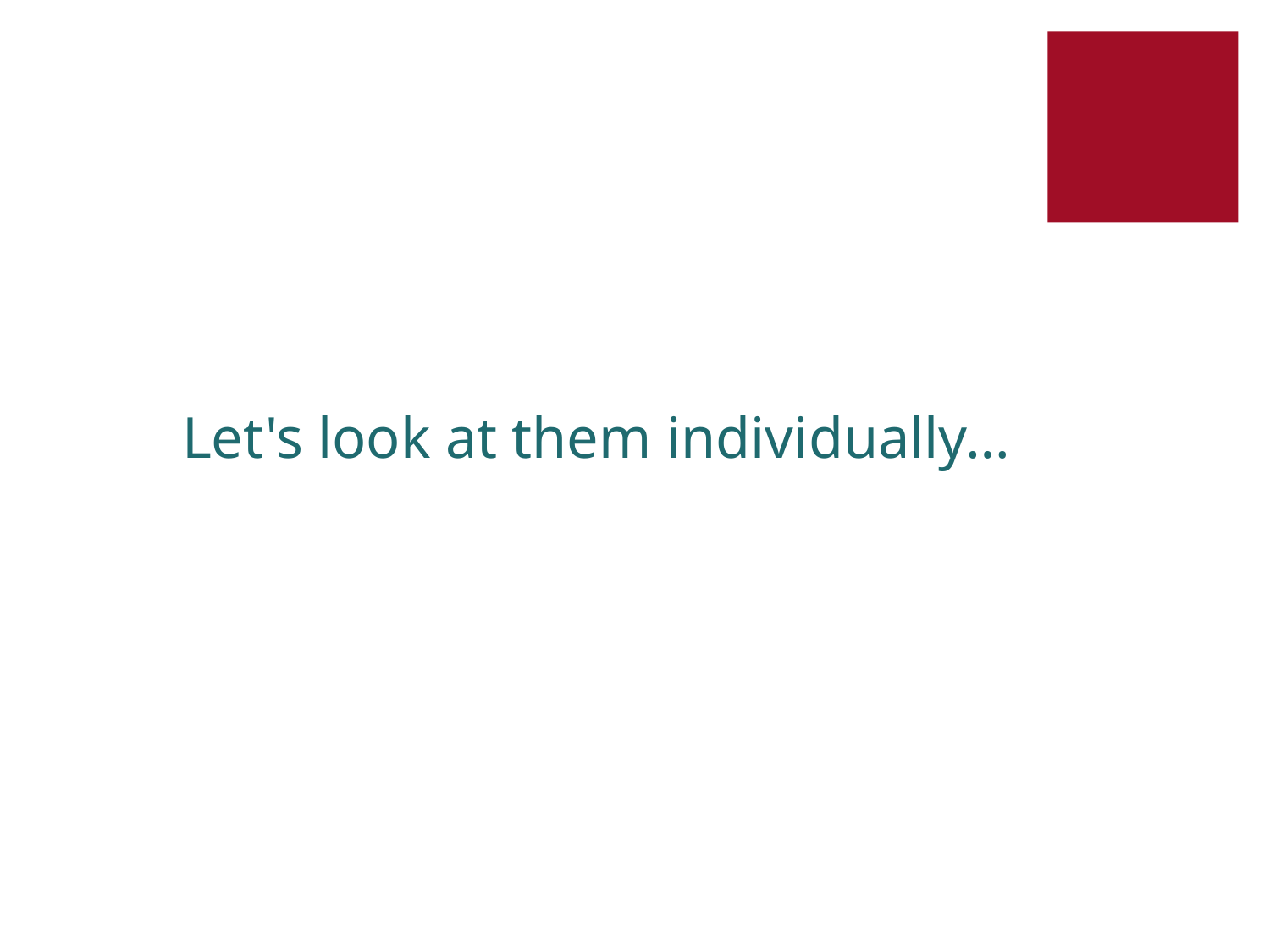

# Let's look at them individually…

## Slide 7
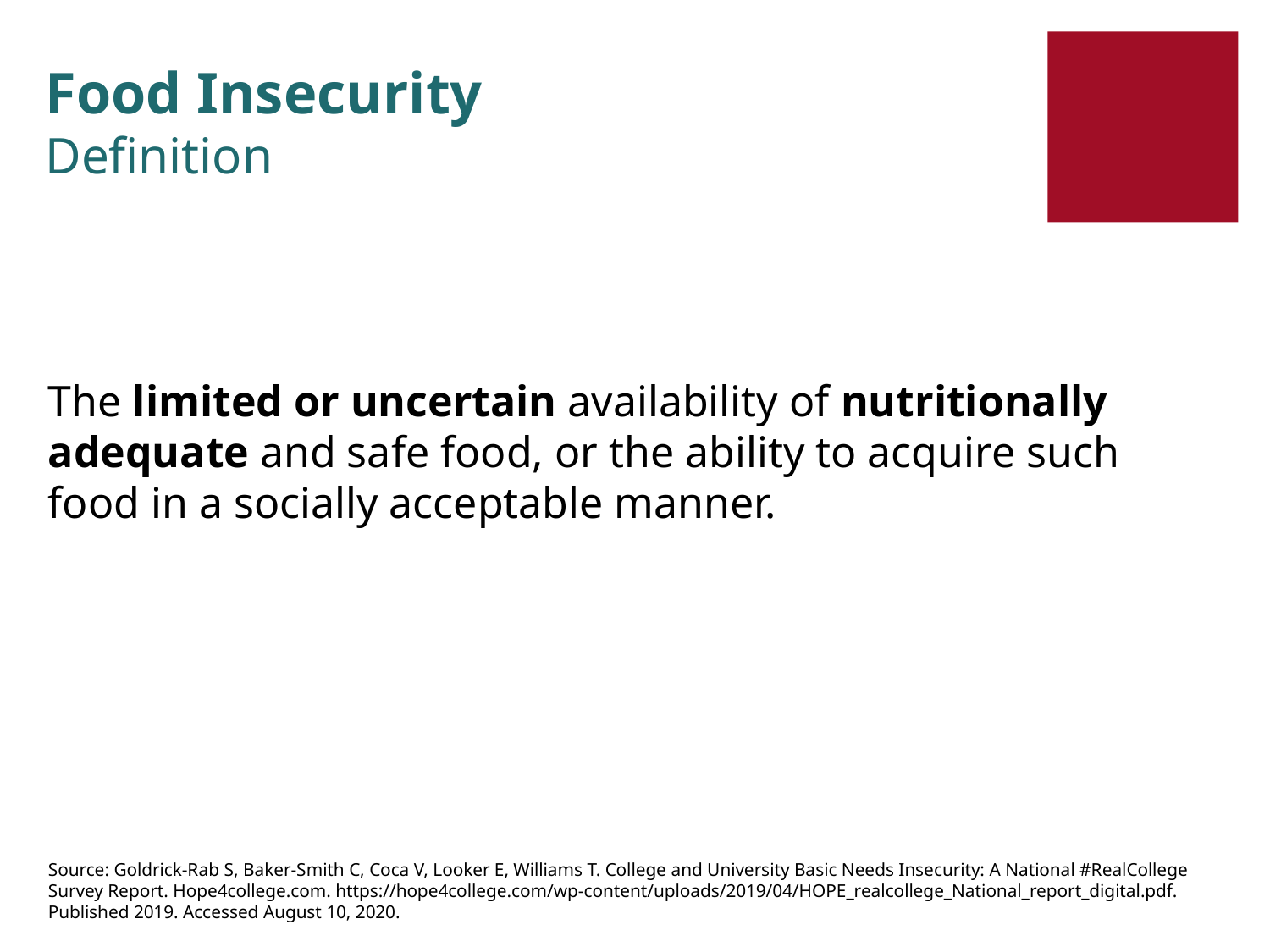

# Food Insecurity Definition
The limited or uncertain availability of nutritionally adequate and safe food, or the ability to acquire such food in a socially acceptable manner.
Source: Goldrick-Rab S, Baker-Smith C, Coca V, Looker E, Williams T. College and University Basic Needs Insecurity: A National #RealCollege Survey Report. Hope4college.com. https://hope4college.com/wp-content/uploads/2019/04/HOPE_realcollege_National_report_digital.pdf. Published 2019. Accessed August 10, 2020.

## Slide 8
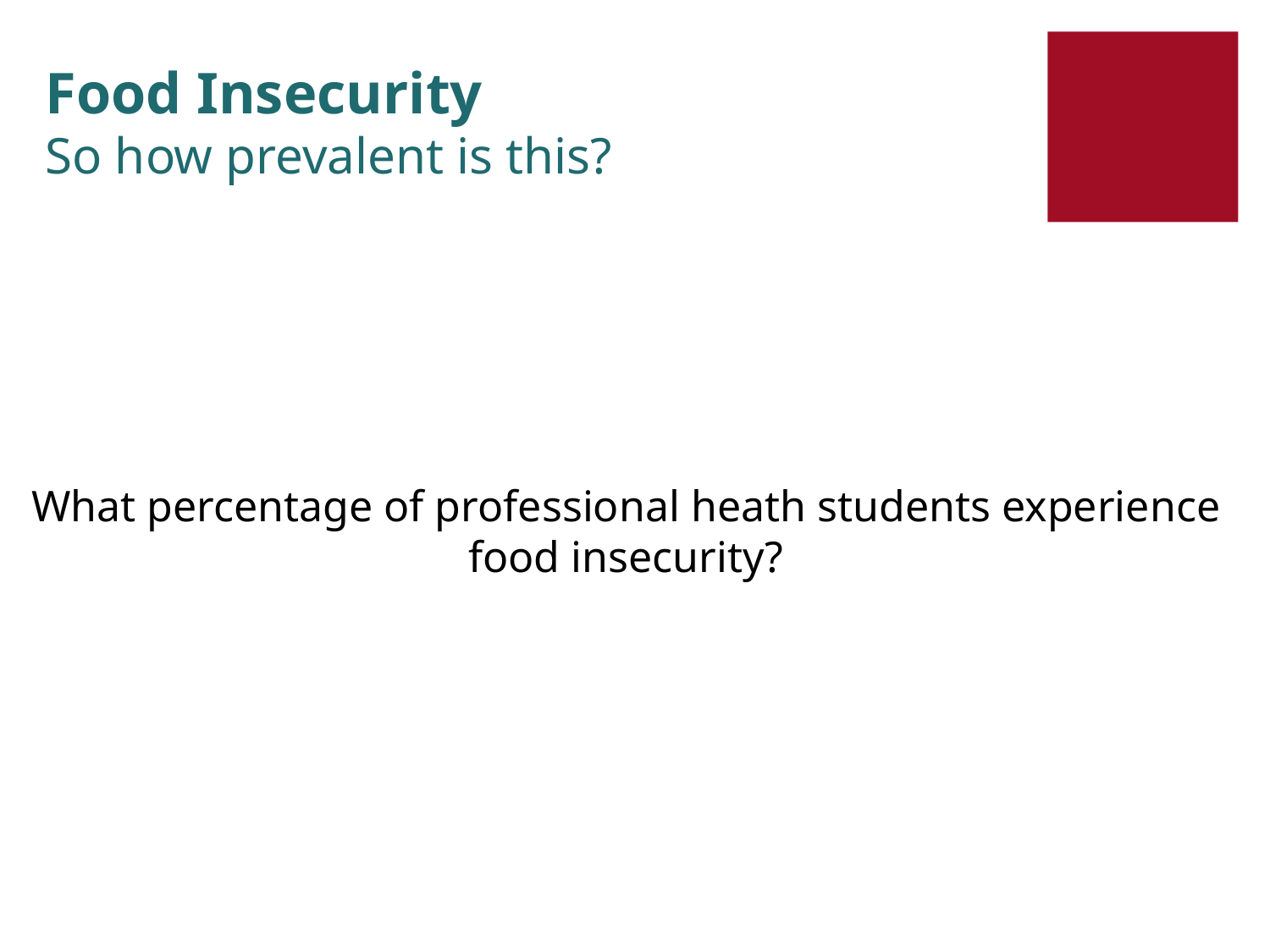

# Food Insecurity So how prevalent is this?
What percentage of professional heath students experience food insecurity?

## Slide 9
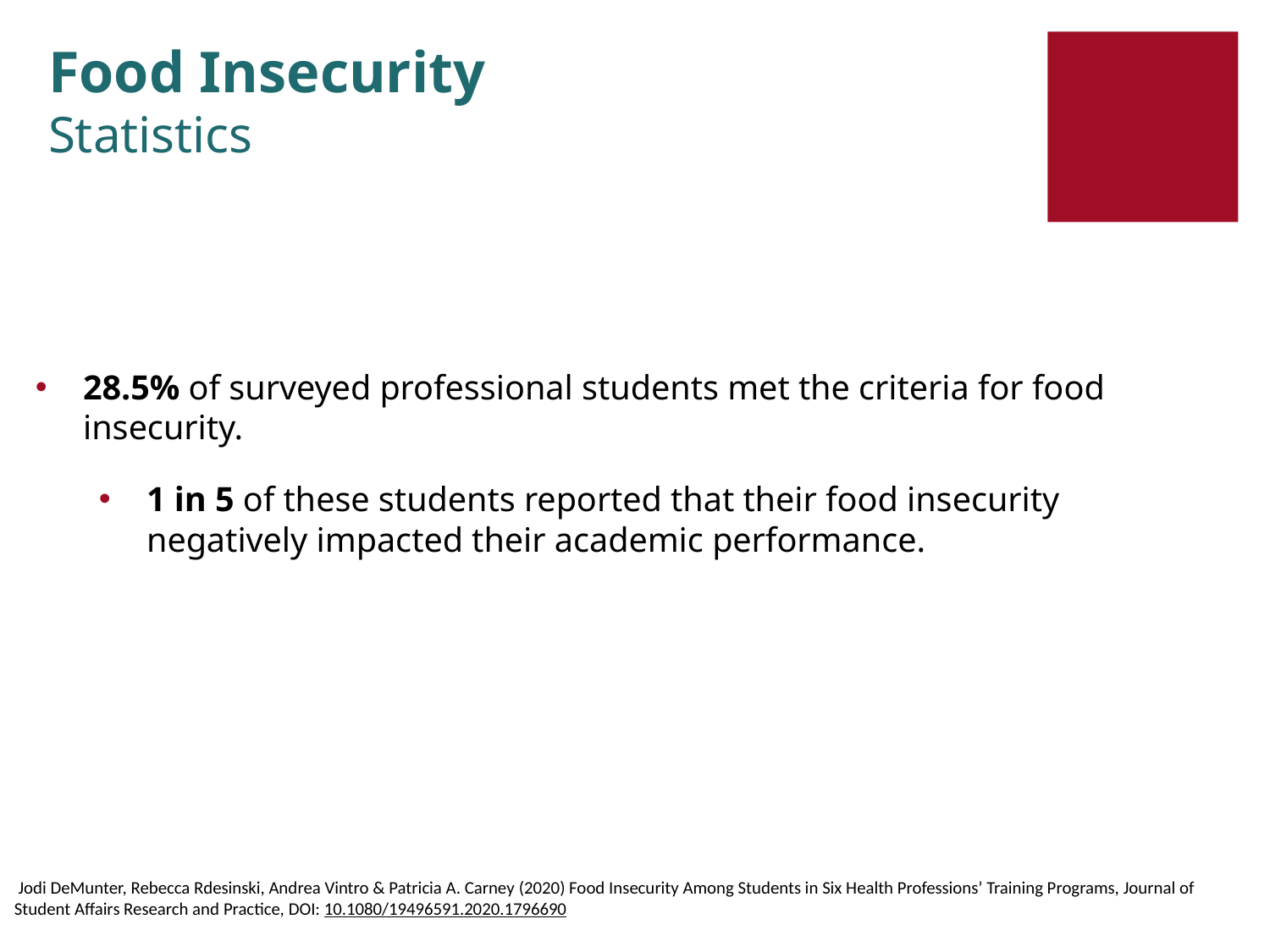

# Food Insecurity Statistics
28.5% of surveyed professional students met the criteria for food insecurity.
1 in 5 of these students reported that their food insecurity negatively impacted their academic performance.
 Jodi DeMunter, Rebecca Rdesinski, Andrea Vintro & Patricia A. Carney (2020) Food Insecurity Among Students in Six Health Professions’ Training Programs, Journal of Student Affairs Research and Practice, DOI: 10.1080/19496591.2020.1796690

## Slide 10
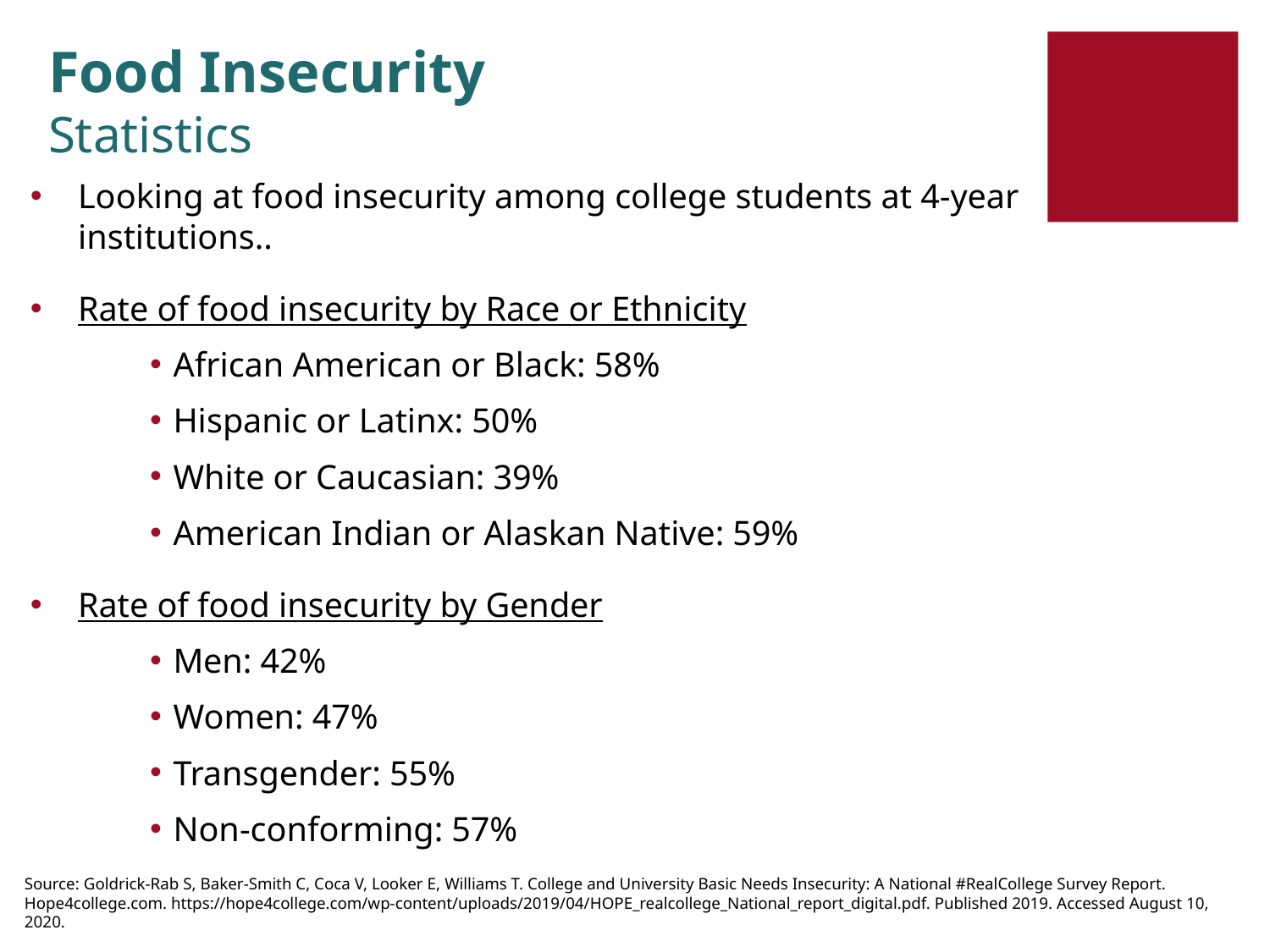

# Food Insecurity Statistics
Looking at food insecurity among college students at 4-year institutions..
Rate of food insecurity by Race or Ethnicity
African American or Black: 58%
Hispanic or Latinx: 50%
White or Caucasian: 39%
American Indian or Alaskan Native: 59%
Rate of food insecurity by Gender
Men: 42%
Women: 47%
Transgender: 55%
Non-conforming: 57%
Source: Goldrick-Rab S, Baker-Smith C, Coca V, Looker E, Williams T. College and University Basic Needs Insecurity: A National #RealCollege Survey Report. Hope4college.com. https://hope4college.com/wp-content/uploads/2019/04/HOPE_realcollege_National_report_digital.pdf. Published 2019. Accessed August 10, 2020.

## Slide 11
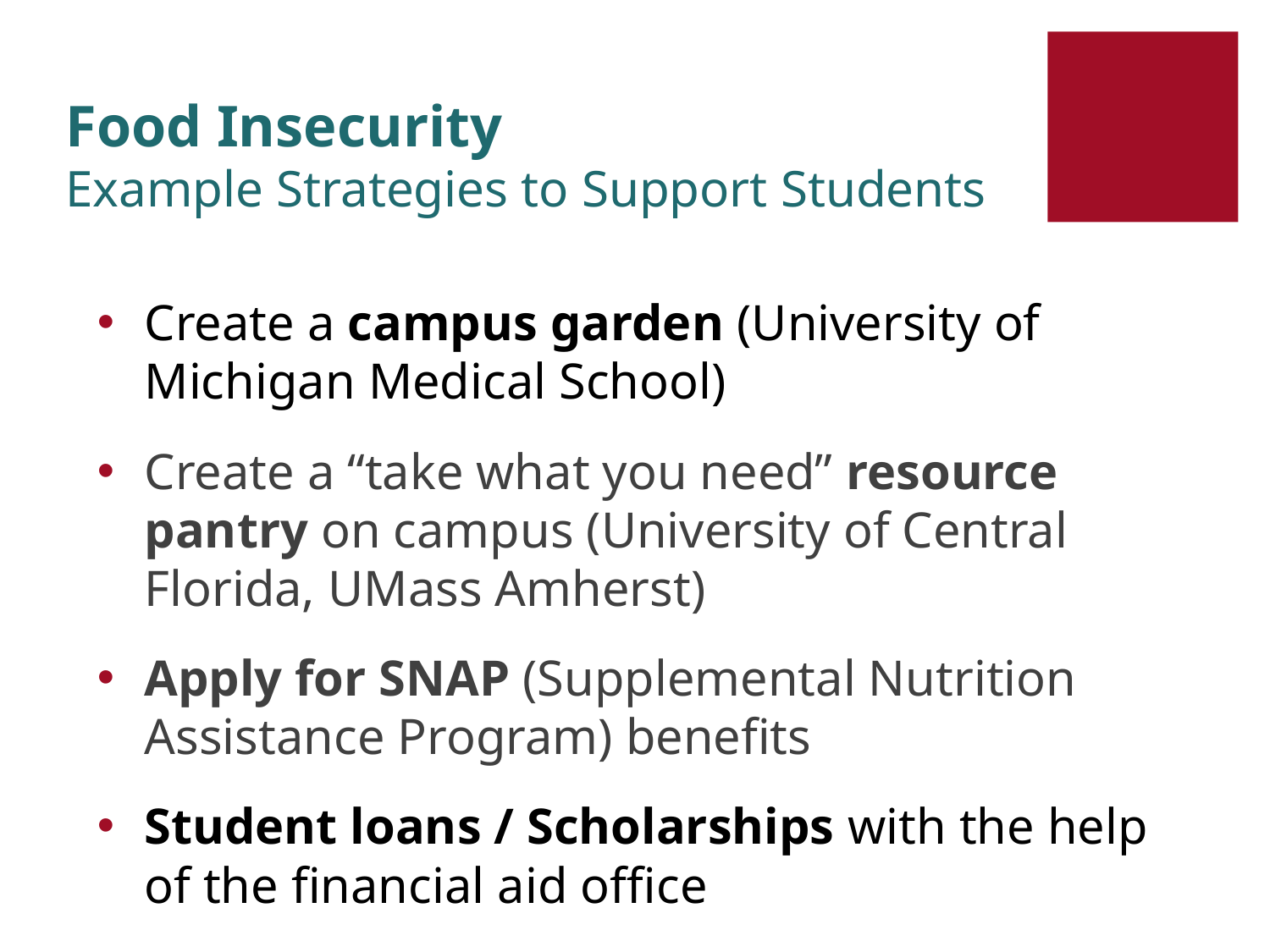

# Food Insecurity Example Strategies to Support Students
Create a campus garden (University of Michigan Medical School)
Create a “take what you need” resource pantry on campus (University of Central Florida, UMass Amherst)
Apply for SNAP (Supplemental Nutrition Assistance Program) benefits
Student loans / Scholarships with the help of the financial aid office

## Slide 12
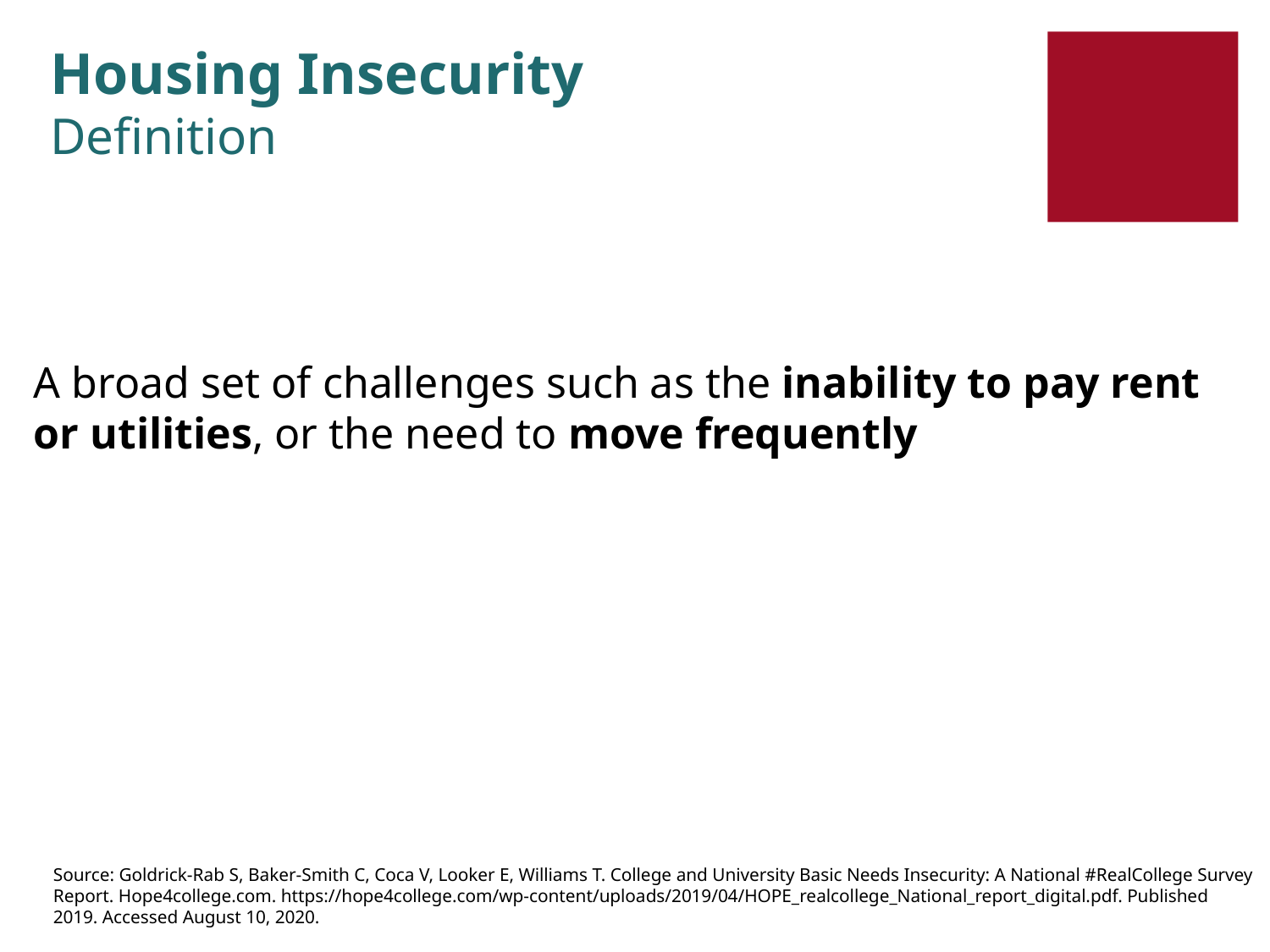

# Housing Insecurity Definition
A broad set of challenges such as the inability to pay rent or utilities, or the need to move frequently
Source: Goldrick-Rab S, Baker-Smith C, Coca V, Looker E, Williams T. College and University Basic Needs Insecurity: A National #RealCollege Survey Report. Hope4college.com. https://hope4college.com/wp-content/uploads/2019/04/HOPE_realcollege_National_report_digital.pdf. Published 2019. Accessed August 10, 2020.

## Slide 13
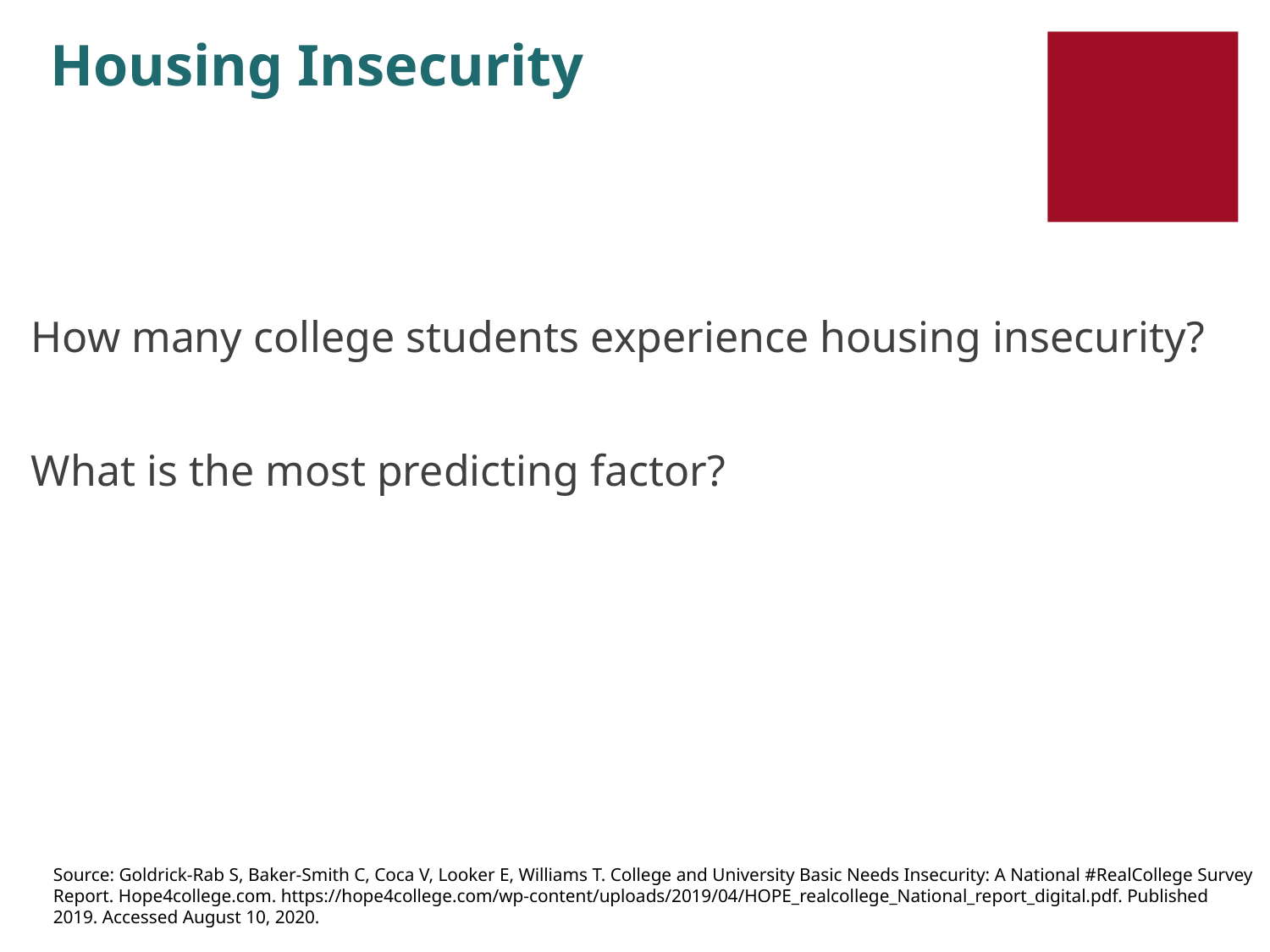

# Housing Insecurity
How many college students experience housing insecurity?
What is the most predicting factor?
Source: Goldrick-Rab S, Baker-Smith C, Coca V, Looker E, Williams T. College and University Basic Needs Insecurity: A National #RealCollege Survey Report. Hope4college.com. https://hope4college.com/wp-content/uploads/2019/04/HOPE_realcollege_National_report_digital.pdf. Published 2019. Accessed August 10, 2020.

## Slide 14
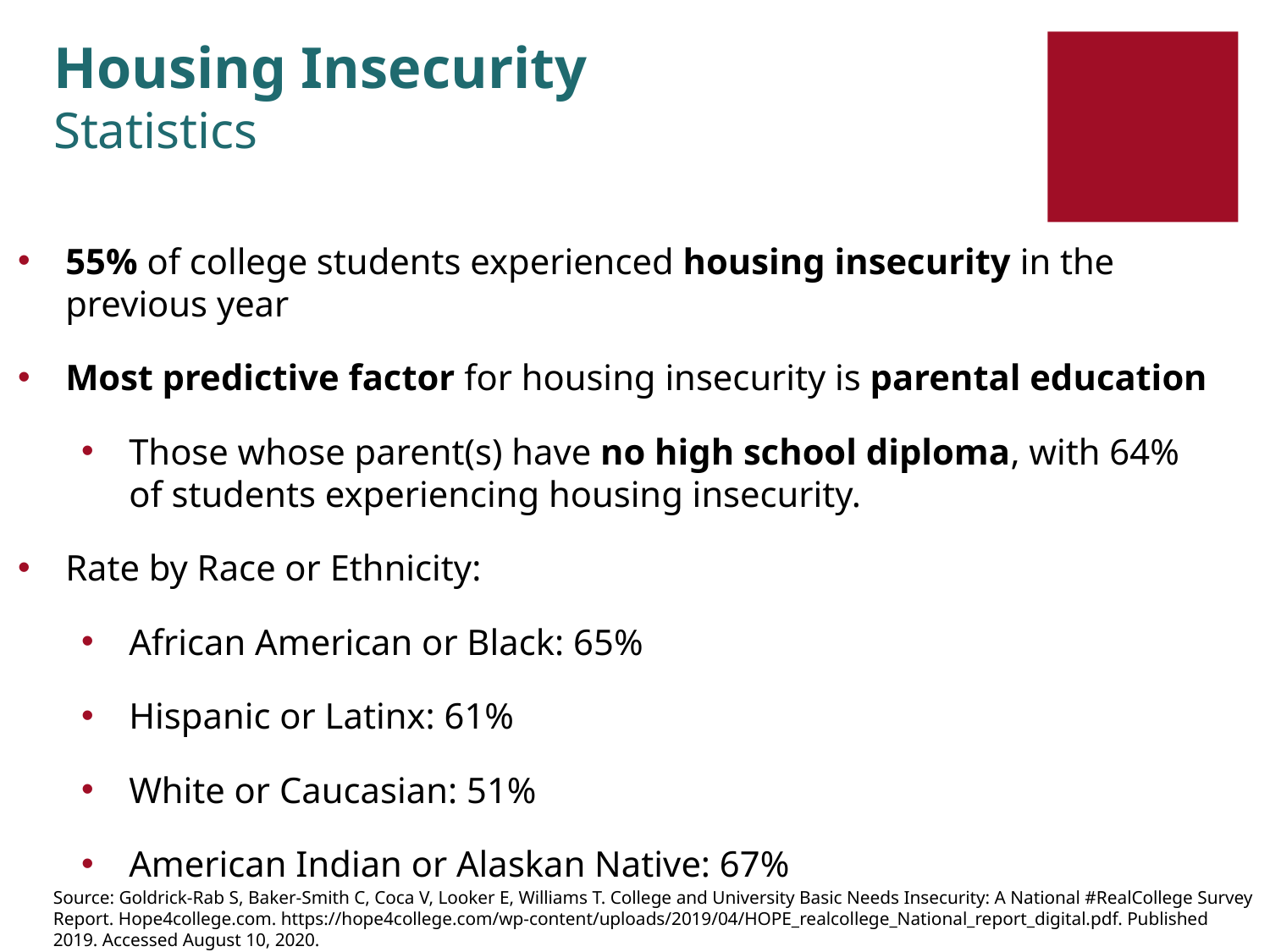

# Housing Insecurity Statistics
55% of college students experienced housing insecurity in the previous year
Most predictive factor for housing insecurity is parental education
Those whose parent(s) have no high school diploma, with 64% of students experiencing housing insecurity.
Rate by Race or Ethnicity:
African American or Black: 65%
Hispanic or Latinx: 61%
White or Caucasian: 51%
American Indian or Alaskan Native: 67%
Source: Goldrick-Rab S, Baker-Smith C, Coca V, Looker E, Williams T. College and University Basic Needs Insecurity: A National #RealCollege Survey Report. Hope4college.com. https://hope4college.com/wp-content/uploads/2019/04/HOPE_realcollege_National_report_digital.pdf. Published 2019. Accessed August 10, 2020.

## Slide 15
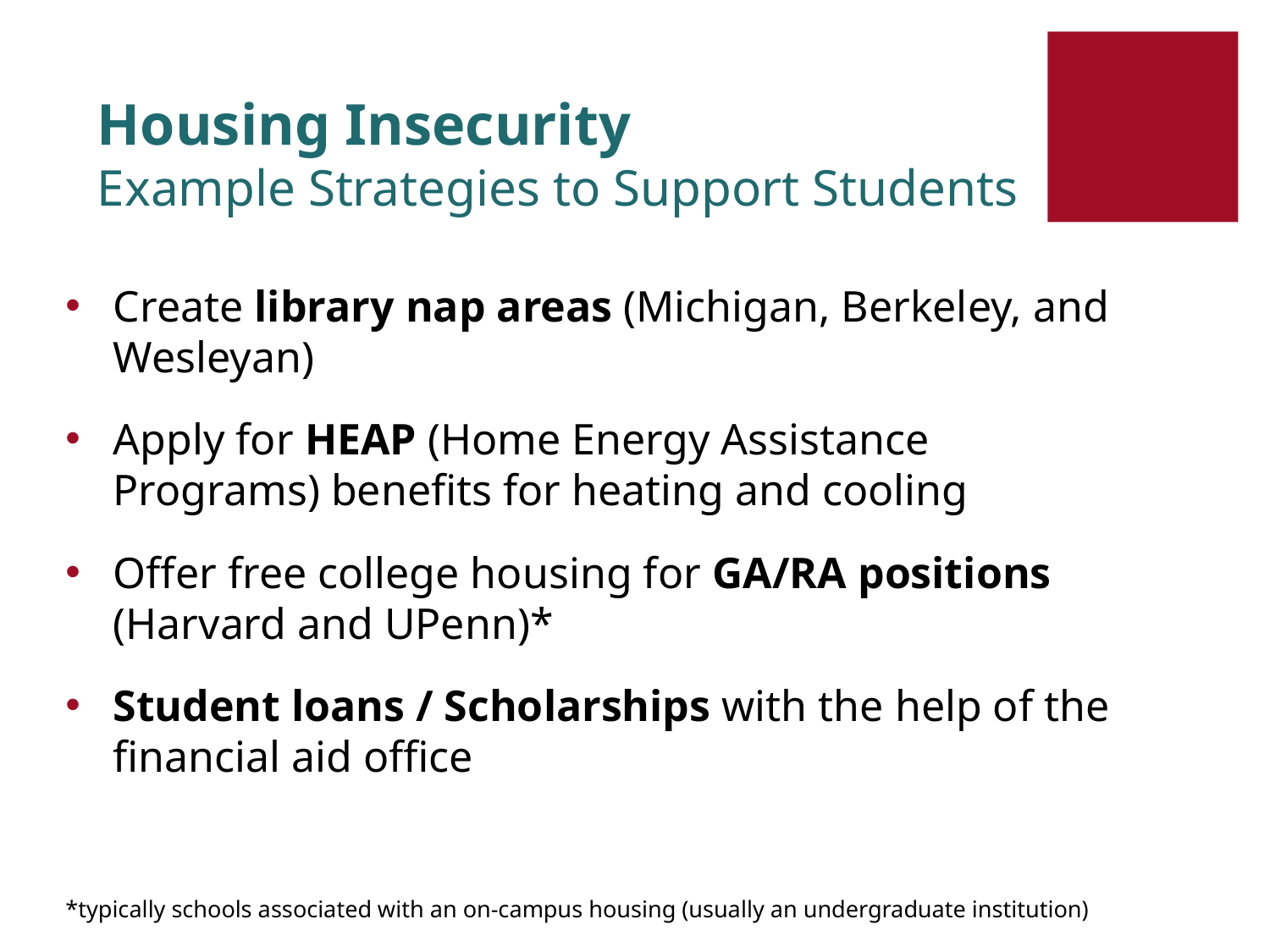

# Housing Insecurity Example Strategies to Support Students
Create library nap areas (Michigan, Berkeley, and Wesleyan)
Apply for HEAP (Home Energy Assistance Programs) benefits for heating and cooling
Offer free college housing for GA/RA positions (Harvard and UPenn)*
Student loans / Scholarships with the help of the financial aid office
*typically schools associated with an on-campus housing (usually an undergraduate institution)

## Slide 16
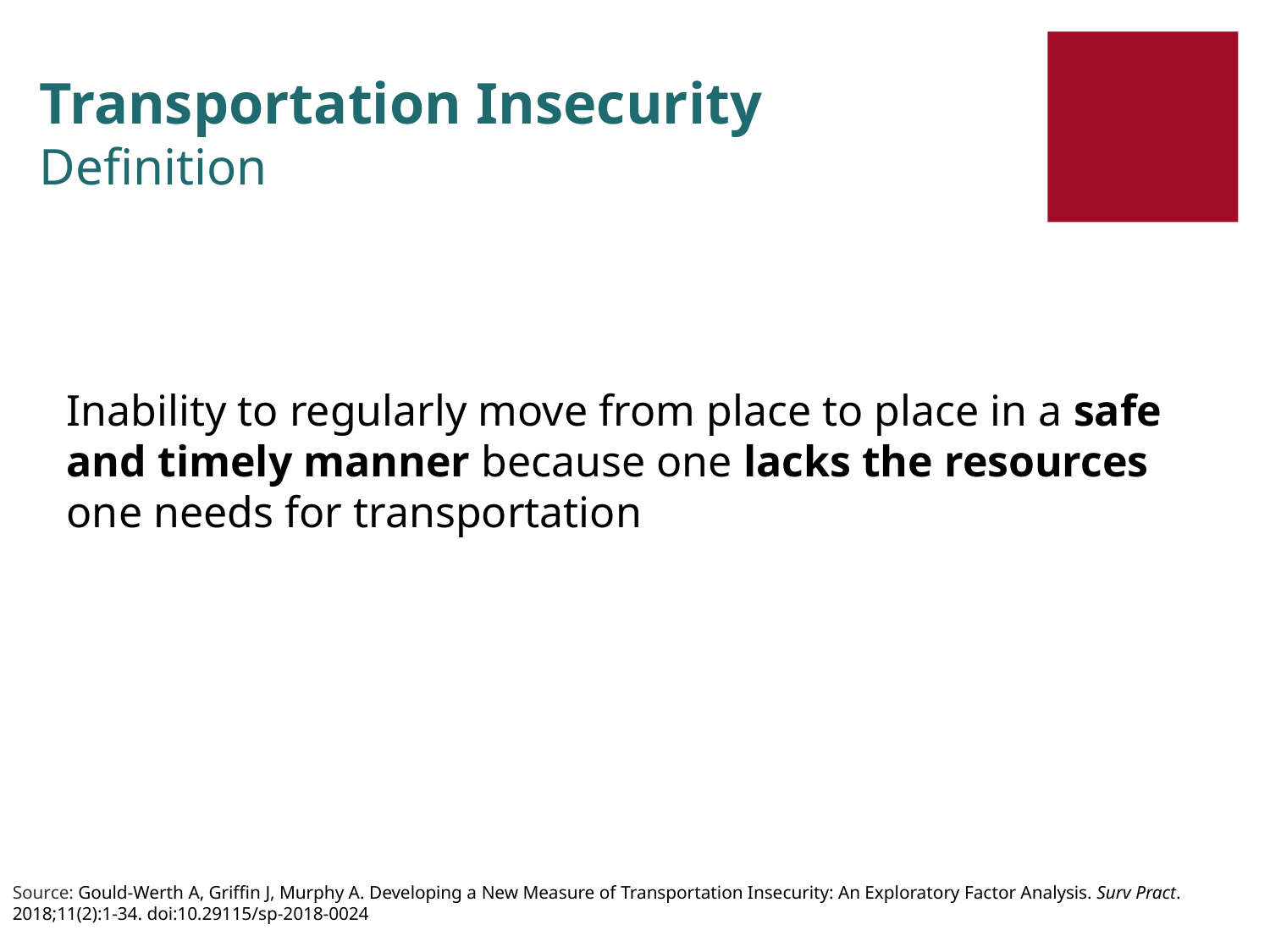

# Transportation Insecurity Definition
Inability to regularly move from place to place in a safe and timely manner because one lacks the resources one needs for transportation
Source: Gould-Werth A, Griffin J, Murphy A. Developing a New Measure of Transportation Insecurity: An Exploratory Factor Analysis. Surv Pract. 2018;11(2):1-34. doi:10.29115/sp-2018-0024

## Slide 17
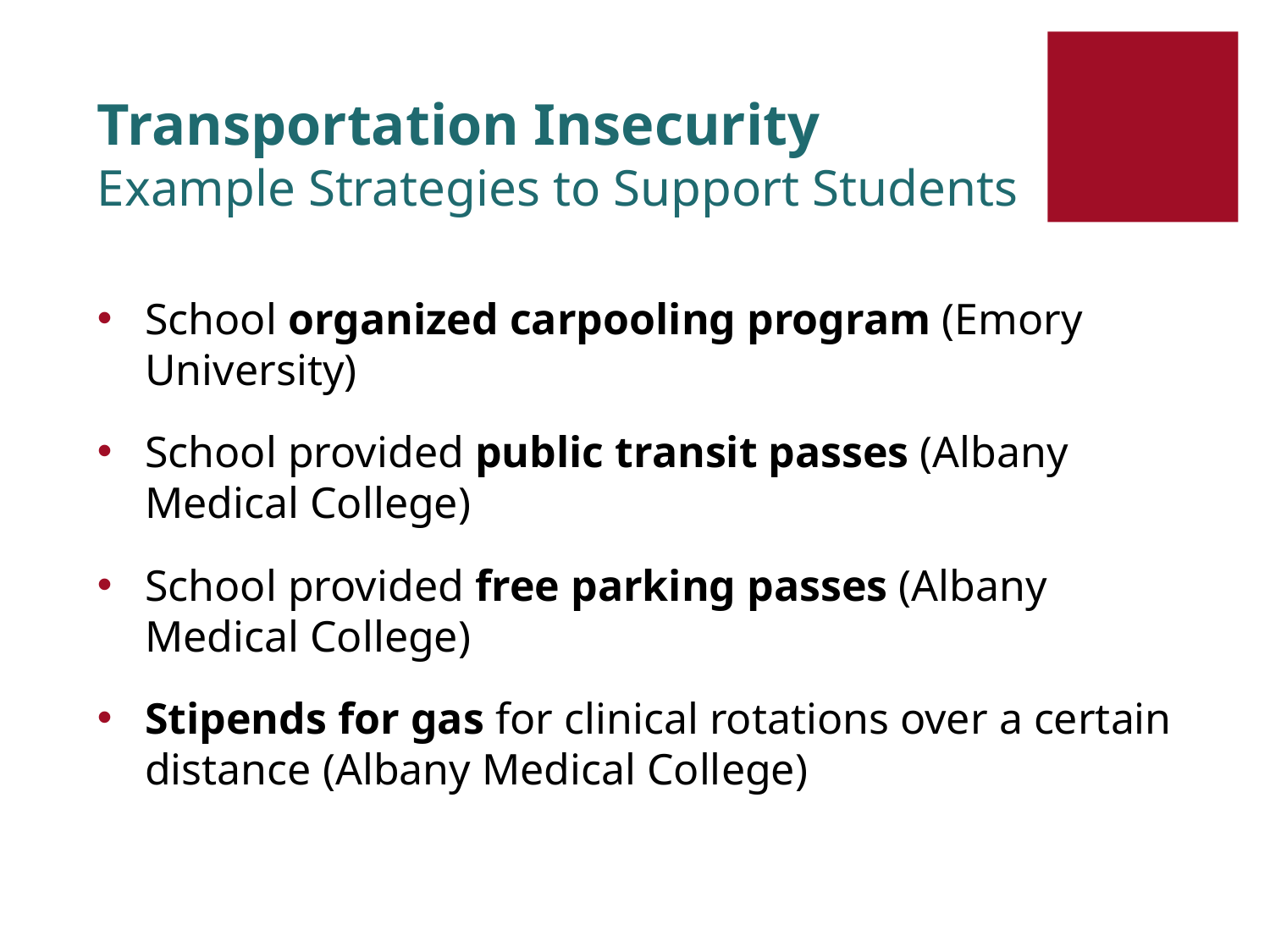

# Transportation Insecurity Example Strategies to Support Students
School organized carpooling program (Emory University)
School provided public transit passes (Albany Medical College)
School provided free parking passes (Albany Medical College)
Stipends for gas for clinical rotations over a certain distance (Albany Medical College)

## Slide 18
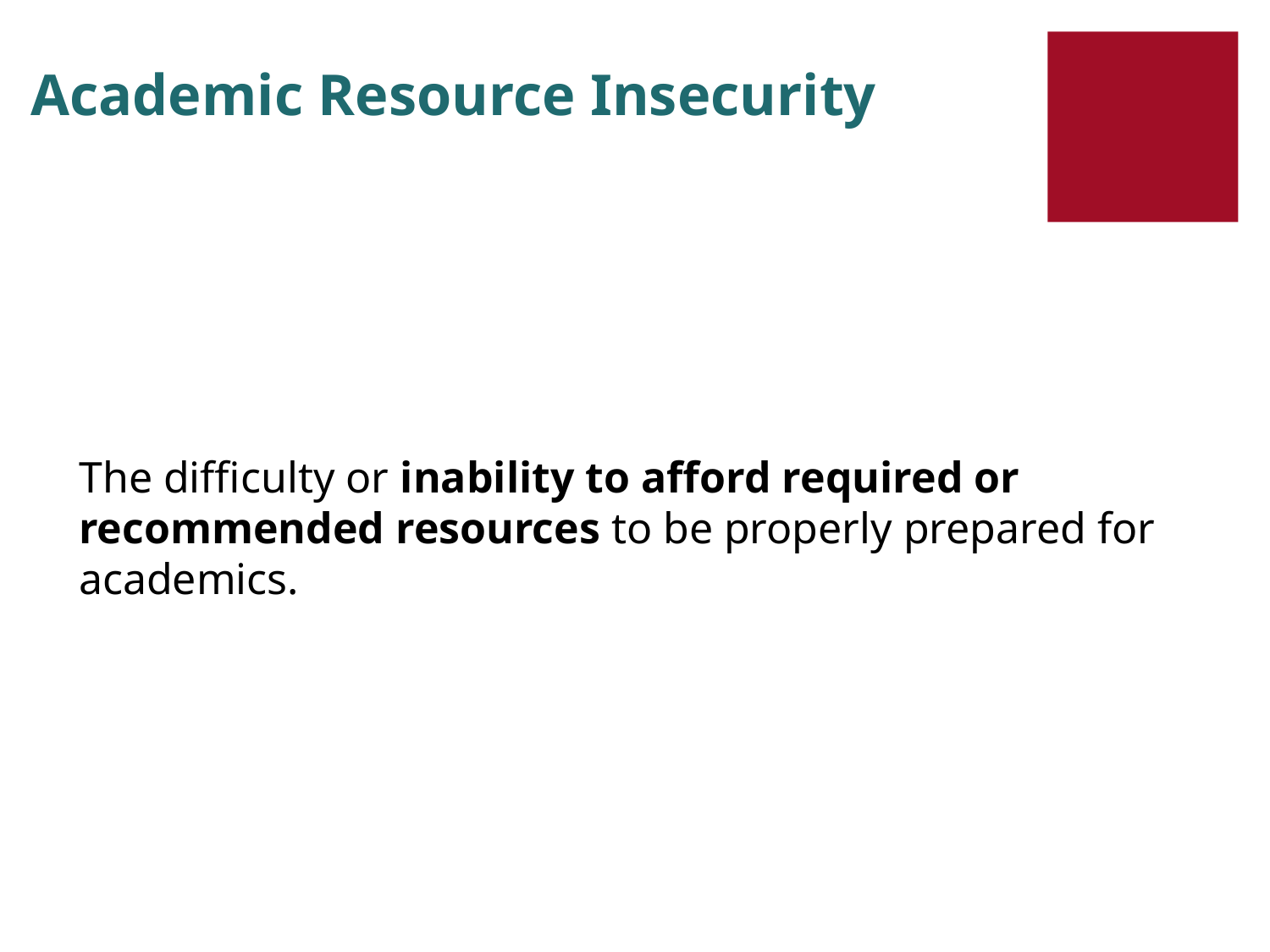

Academic Resource Insecurity
The difficulty or inability to afford required or recommended resources to be properly prepared for academics.

## Slide 19
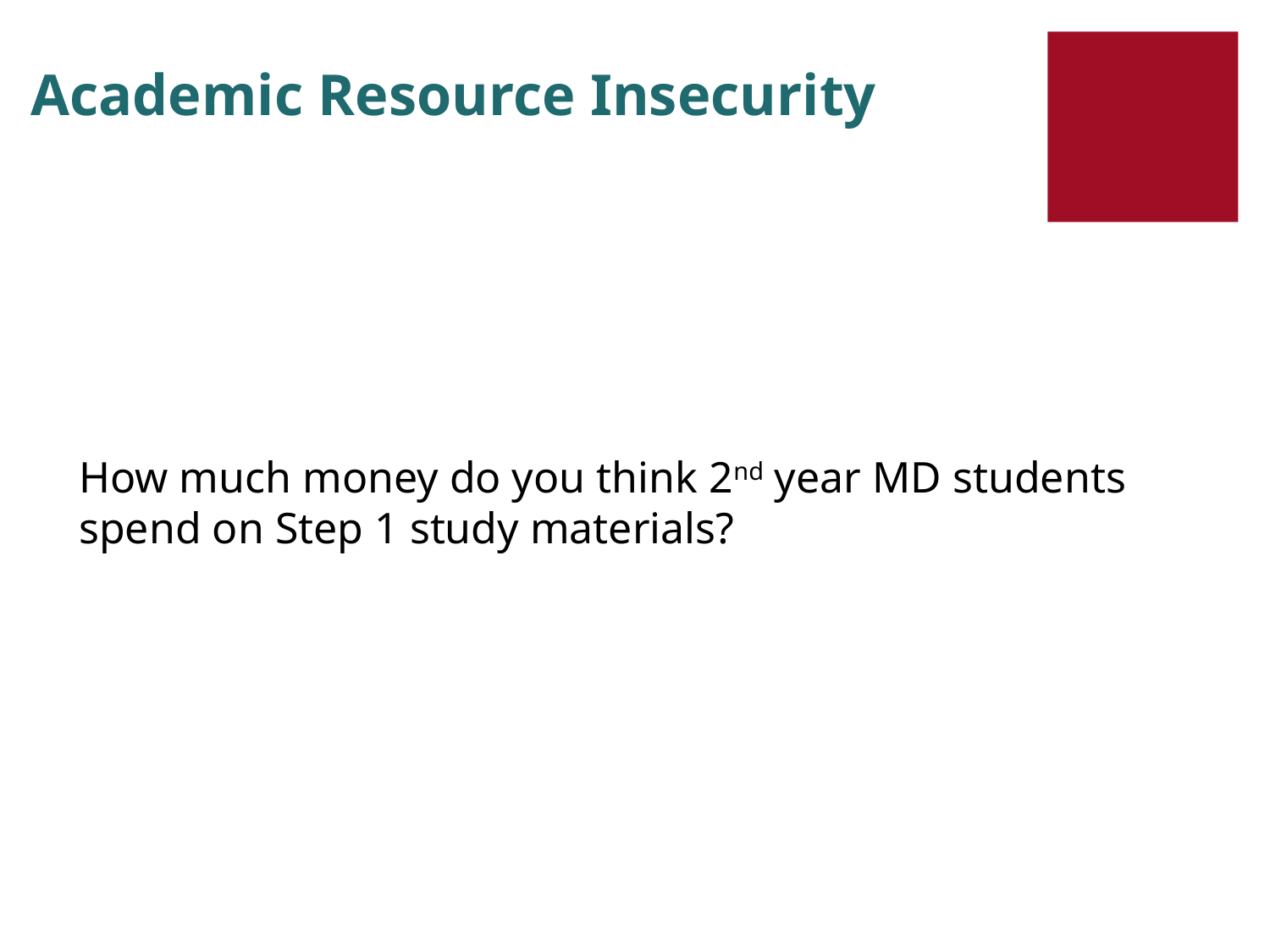

Academic Resource Insecurity
How much money do you think 2nd year MD students spend on Step 1 study materials?

## Slide 20
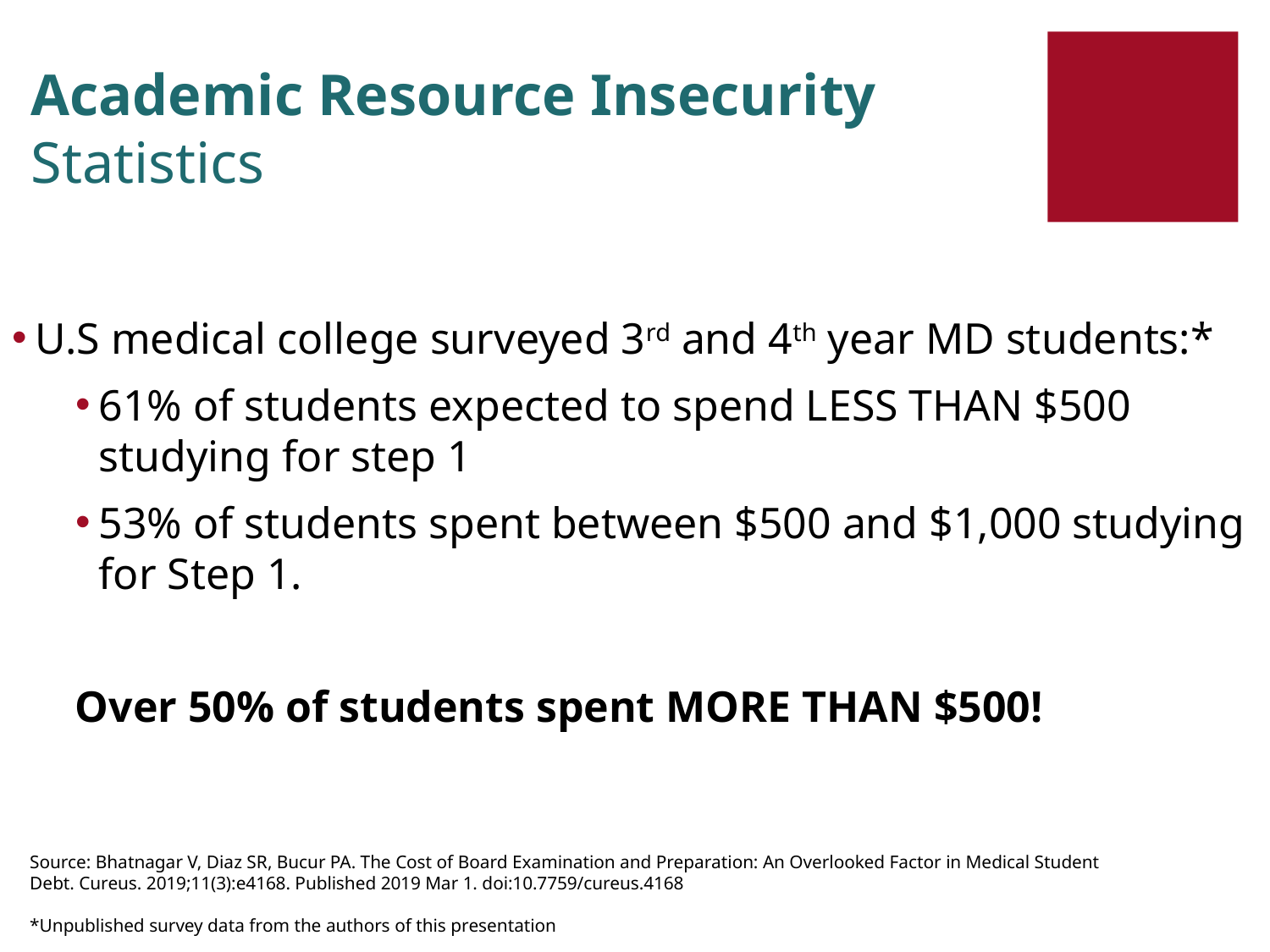

Academic Resource Insecurity
Statistics
U.S medical college surveyed 3rd and 4th year MD students:*
61% of students expected to spend LESS THAN $500 studying for step 1
53% of students spent between $500 and $1,000 studying for Step 1.
Over 50% of students spent MORE THAN $500!
Source: Bhatnagar V, Diaz SR, Bucur PA. The Cost of Board Examination and Preparation: An Overlooked Factor in Medical Student Debt. Cureus. 2019;11(3):e4168. Published 2019 Mar 1. doi:10.7759/cureus.4168
*Unpublished survey data from the authors of this presentation

## Slide 21
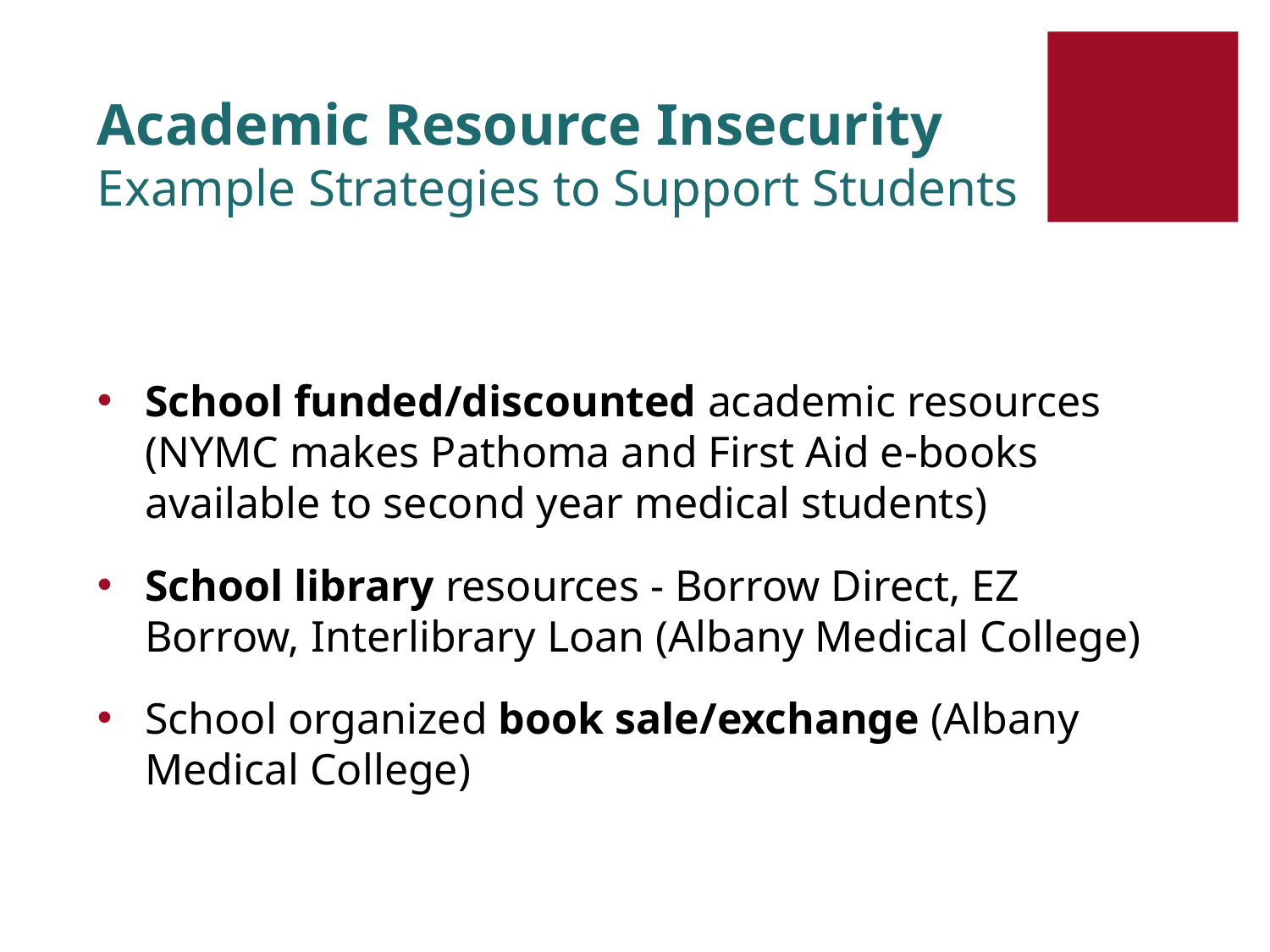

# Academic Resource Insecurity Example Strategies to Support Students
School funded/discounted academic resources (NYMC makes Pathoma and First Aid e-books available to second year medical students)
School library resources - Borrow Direct, EZ Borrow, Interlibrary Loan (Albany Medical College)
School organized book sale/exchange (Albany Medical College)

## Slide 22
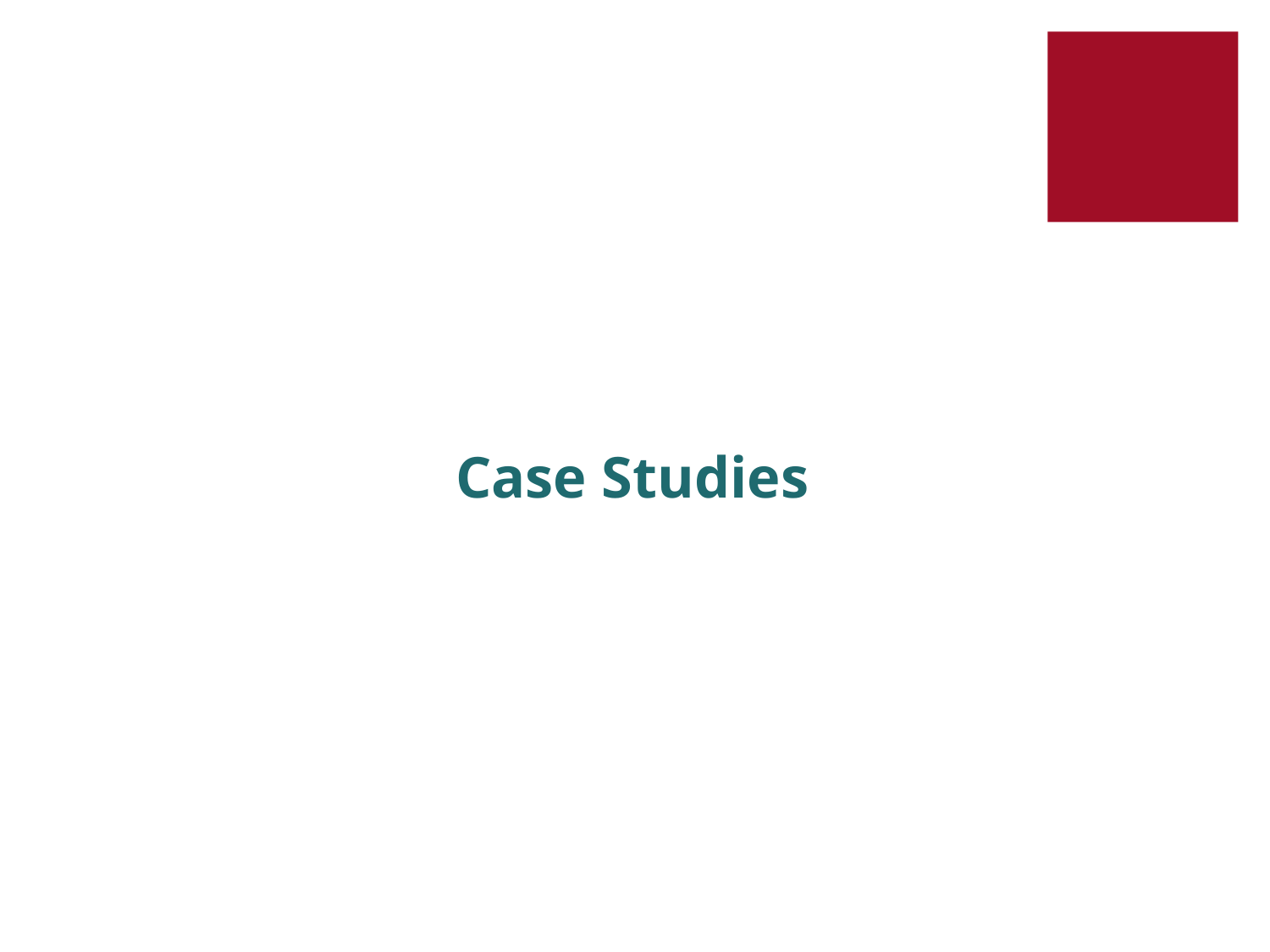

Case Studies

## Slide 23
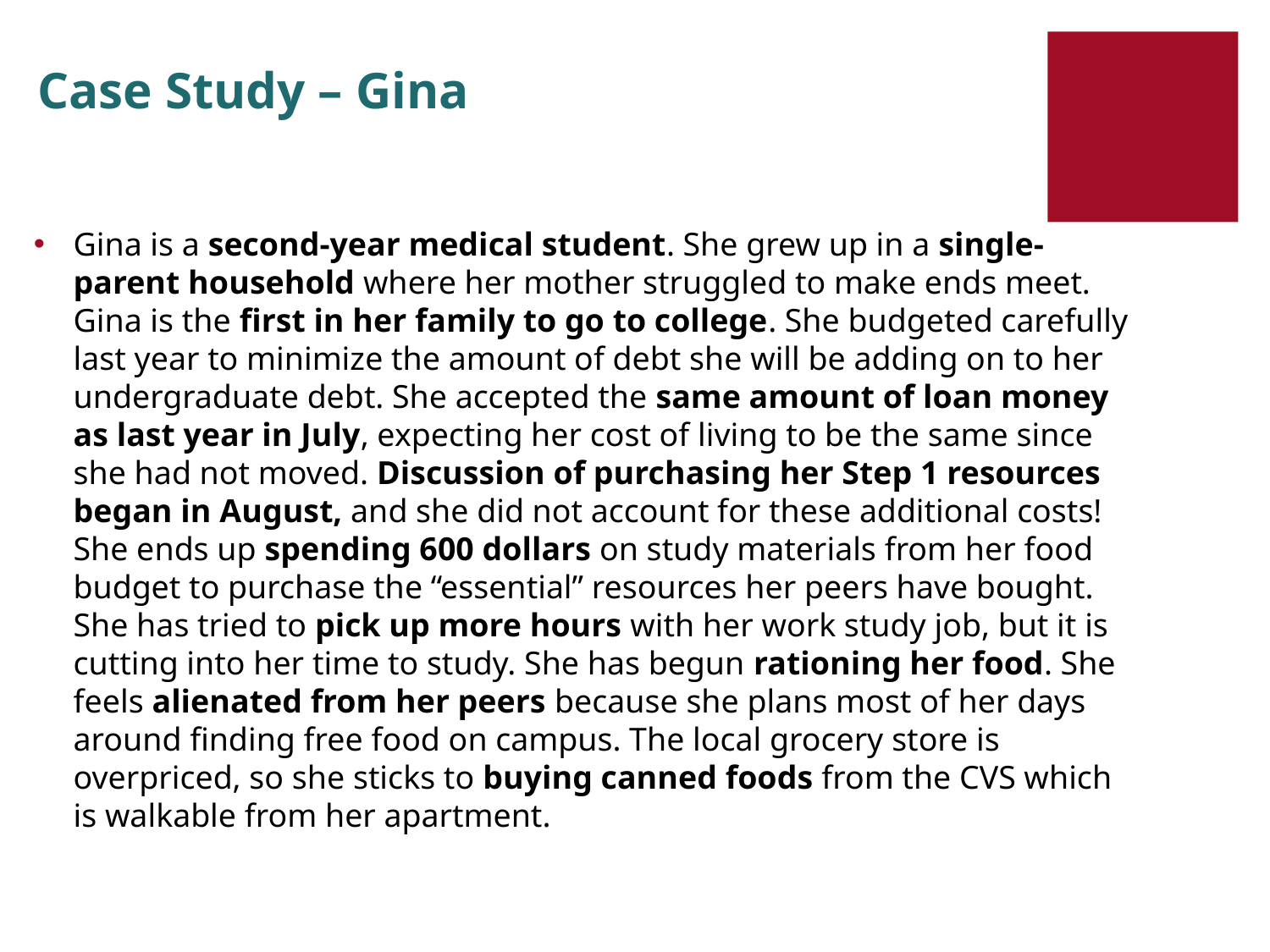

Case Study – Gina
Gina is a second-year medical student. She grew up in a single-parent household where her mother struggled to make ends meet. Gina is the first in her family to go to college. She budgeted carefully last year to minimize the amount of debt she will be adding on to her undergraduate debt. She accepted the same amount of loan money as last year in July, expecting her cost of living to be the same since she had not moved. Discussion of purchasing her Step 1 resources began in August, and she did not account for these additional costs! She ends up spending 600 dollars on study materials from her food budget to purchase the “essential” resources her peers have bought. She has tried to pick up more hours with her work study job, but it is cutting into her time to study. She has begun rationing her food. She feels alienated from her peers because she plans most of her days around finding free food on campus. The local grocery store is overpriced, so she sticks to buying canned foods from the CVS which is walkable from her apartment.

## Slide 24
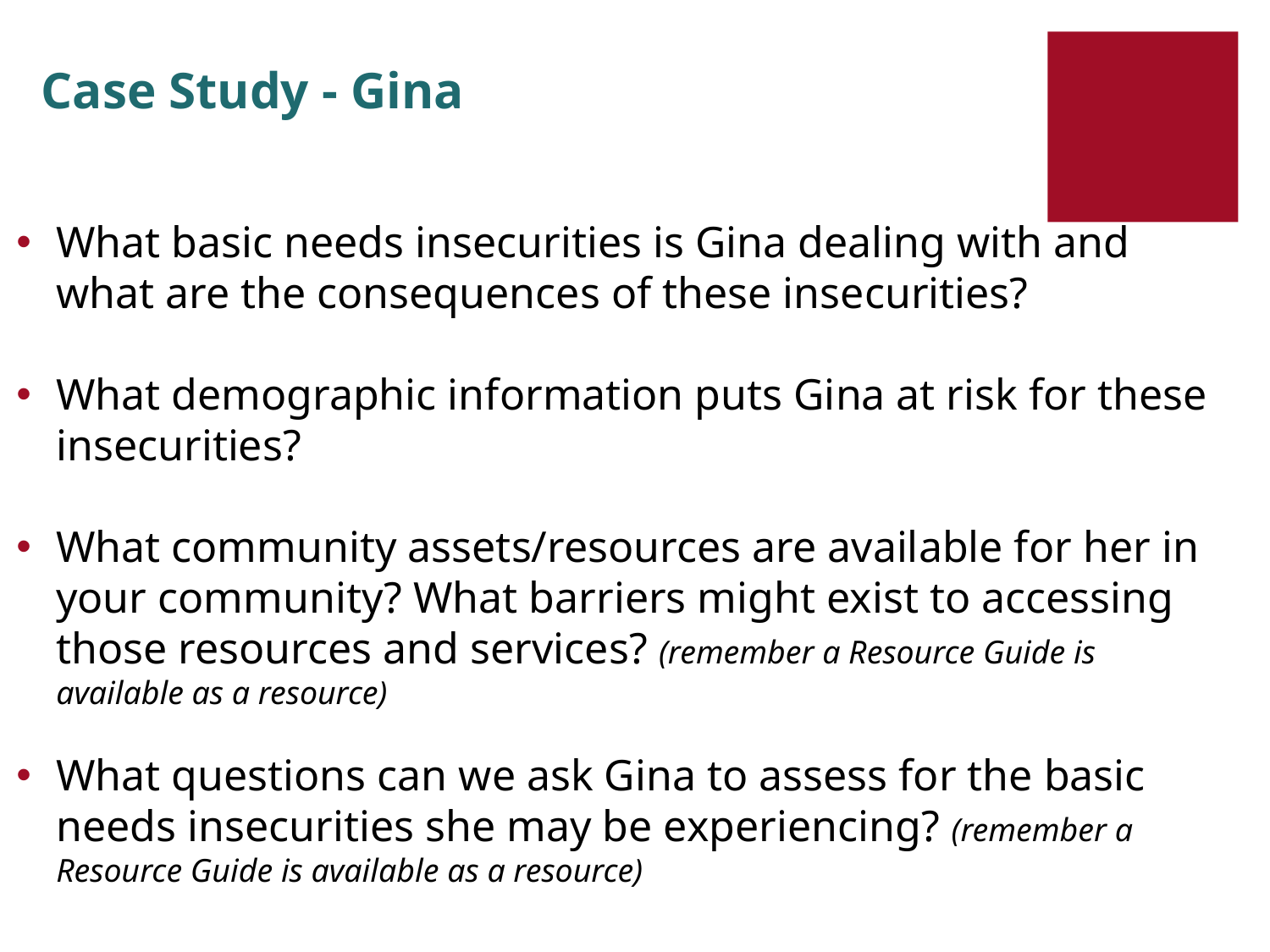

Case Study - Gina
What basic needs insecurities is Gina dealing with and what are the consequences of these insecurities?
What demographic information puts Gina at risk for these insecurities?
What community assets/resources are available for her in your community? What barriers might exist to accessing those resources and services? (remember a Resource Guide is available as a resource)
What questions can we ask Gina to assess for the basic needs insecurities she may be experiencing? (remember a Resource Guide is available as a resource)

## Slide 25
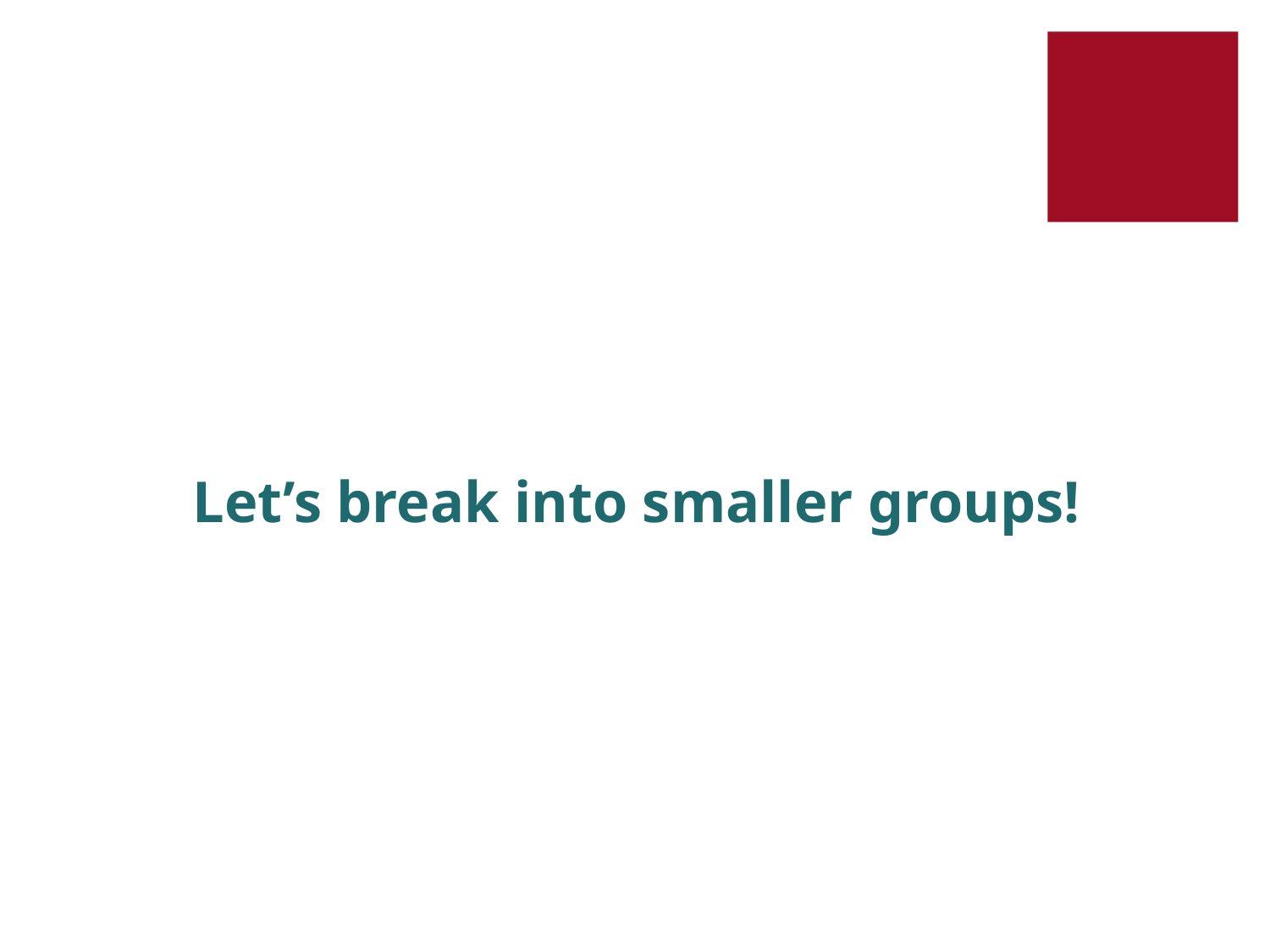

# Let’s break into smaller groups!

## Slide 26
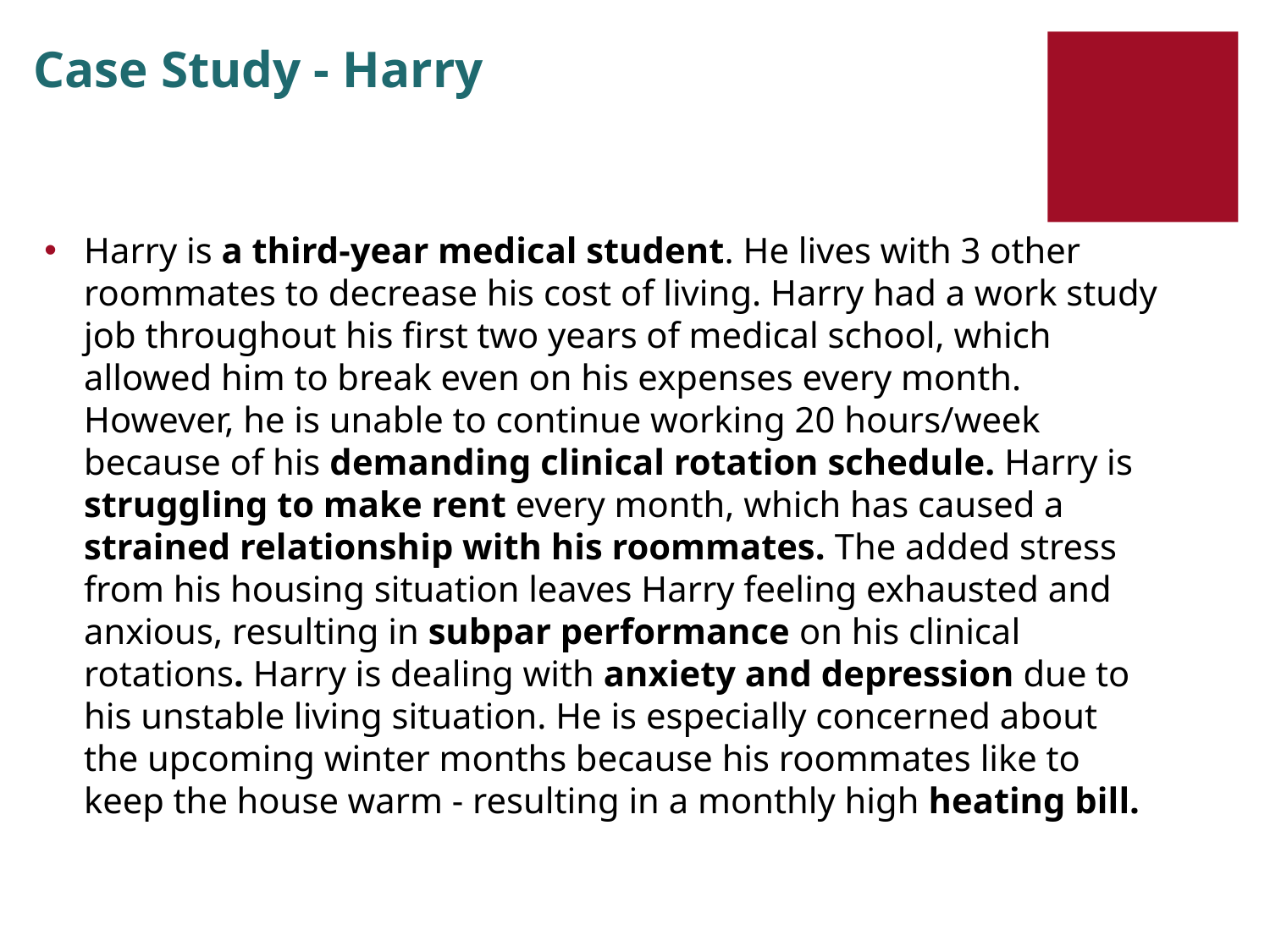

Case Study - Harry
Harry is a third-year medical student. He lives with 3 other roommates to decrease his cost of living. Harry had a work study job throughout his first two years of medical school, which allowed him to break even on his expenses every month. However, he is unable to continue working 20 hours/week because of his demanding clinical rotation schedule. Harry is struggling to make rent every month, which has caused a strained relationship with his roommates. The added stress from his housing situation leaves Harry feeling exhausted and anxious, resulting in subpar performance on his clinical rotations. Harry is dealing with anxiety and depression due to his unstable living situation. He is especially concerned about the upcoming winter months because his roommates like to keep the house warm - resulting in a monthly high heating bill.

## Slide 27
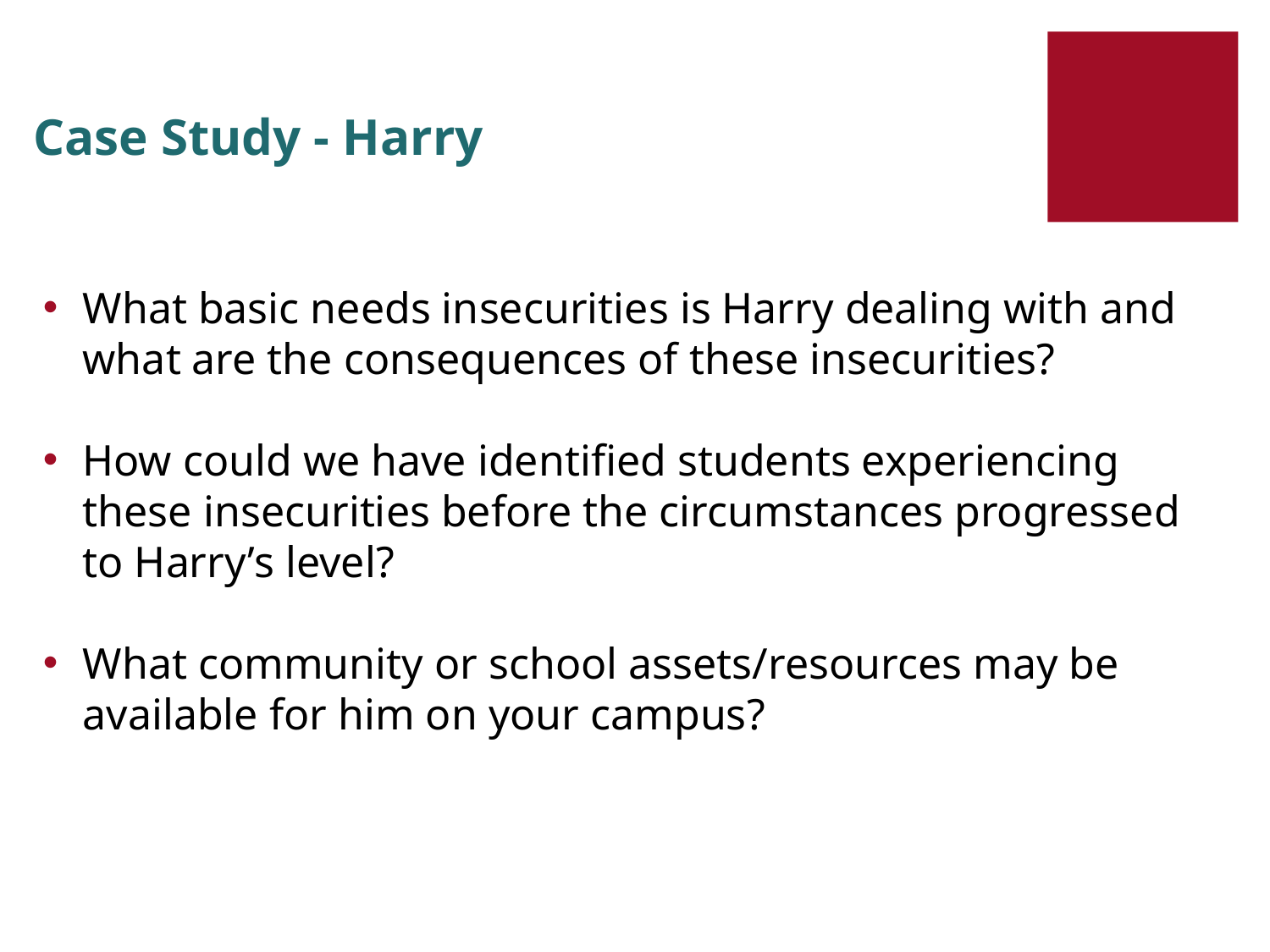

Case Study - Harry
What basic needs insecurities is Harry dealing with and what are the consequences of these insecurities?
How could we have identified students experiencing these insecurities before the circumstances progressed to Harry’s level?
What community or school assets/resources may be available for him on your campus?

## Slide 28
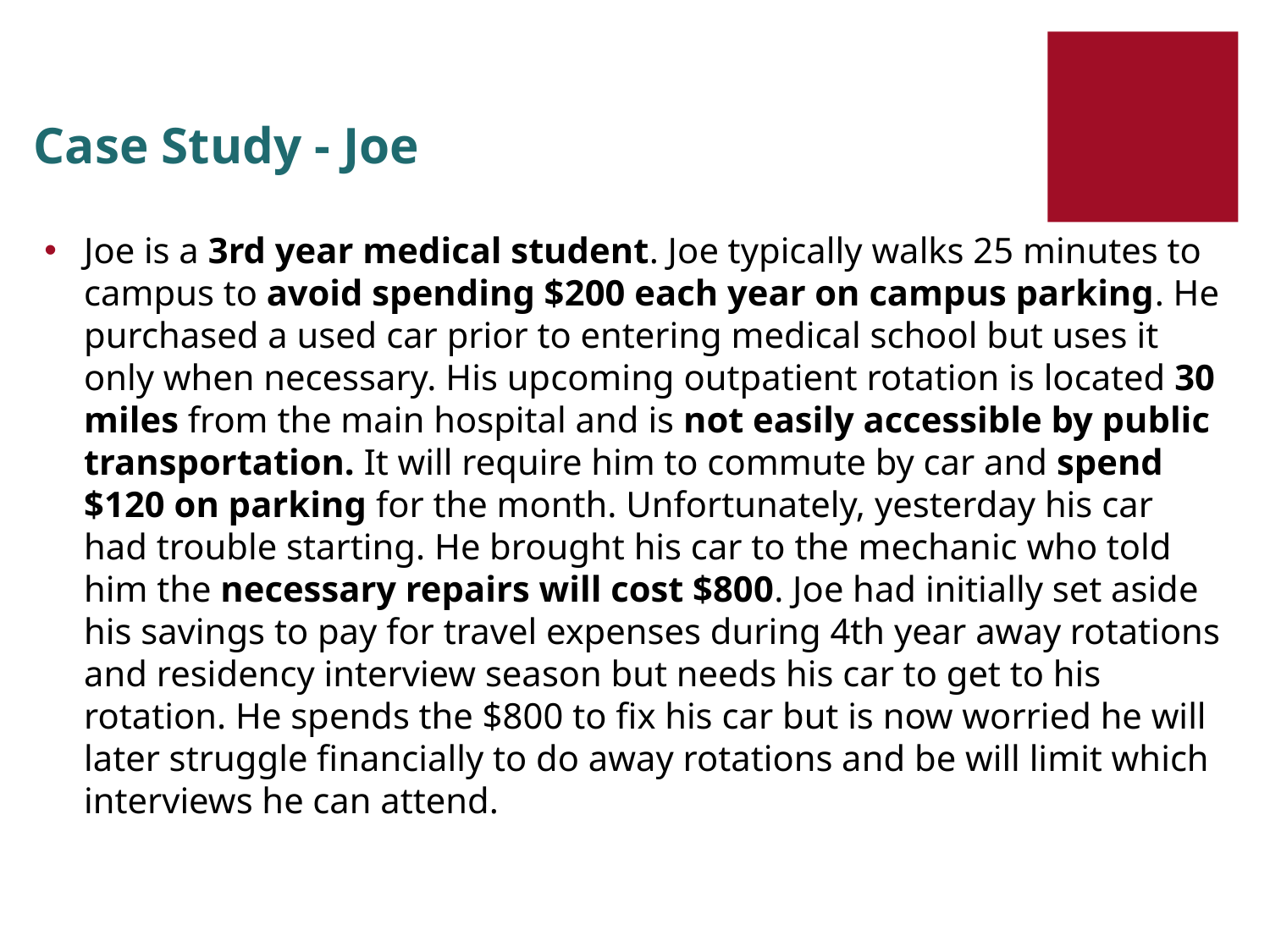

# Case Study - Joe
Joe is a 3rd year medical student. Joe typically walks 25 minutes to campus to avoid spending $200 each year on campus parking. He purchased a used car prior to entering medical school but uses it only when necessary. His upcoming outpatient rotation is located 30 miles from the main hospital and is not easily accessible by public transportation. It will require him to commute by car and spend $120 on parking for the month. Unfortunately, yesterday his car had trouble starting. He brought his car to the mechanic who told him the necessary repairs will cost $800. Joe had initially set aside his savings to pay for travel expenses during 4th year away rotations and residency interview season but needs his car to get to his rotation. He spends the $800 to fix his car but is now worried he will later struggle financially to do away rotations and be will limit which interviews he can attend.

## Slide 29
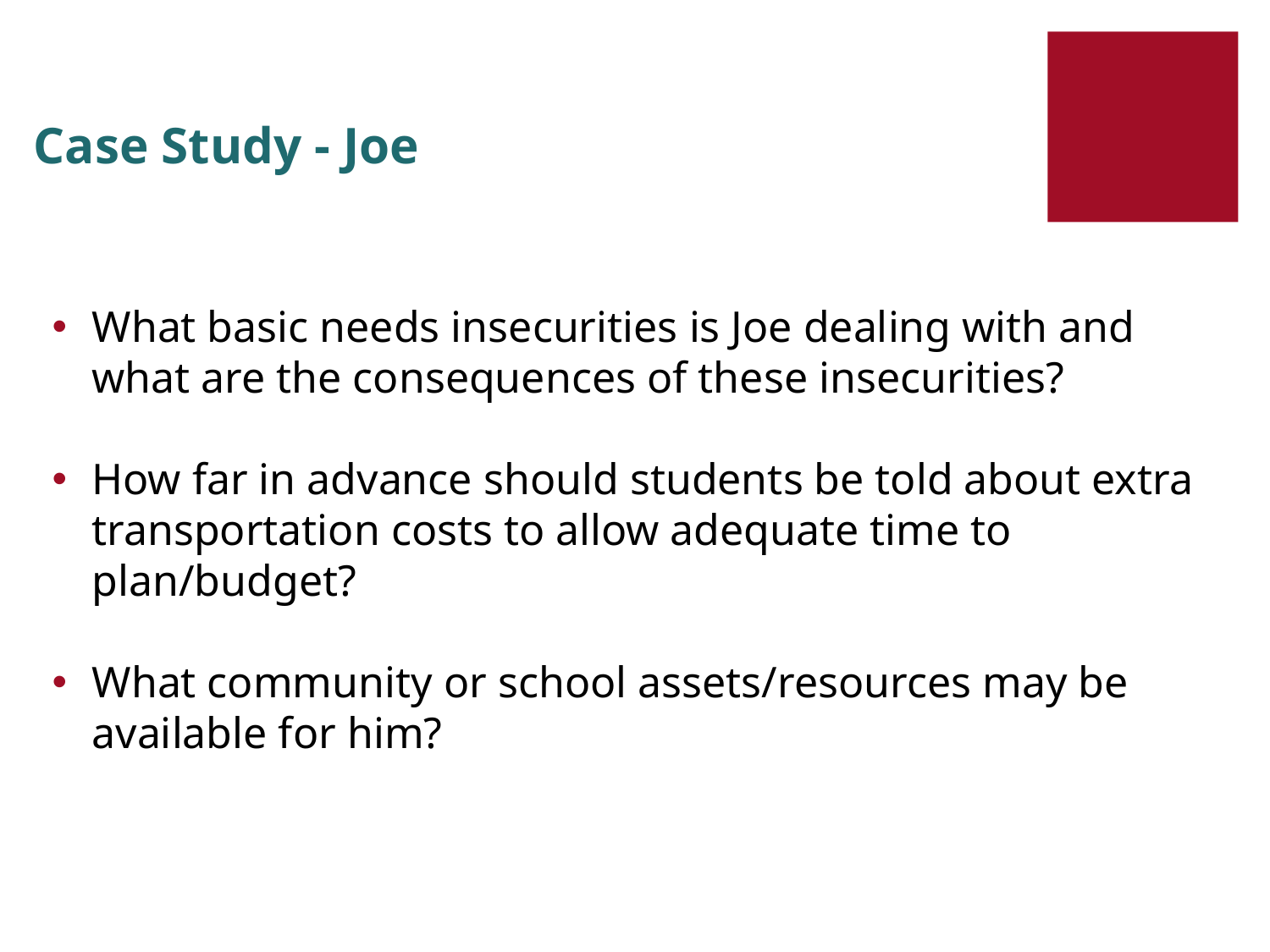

# Case Study - Joe
What basic needs insecurities is Joe dealing with and what are the consequences of these insecurities?
How far in advance should students be told about extra transportation costs to allow adequate time to plan/budget?
What community or school assets/resources may be available for him?

## Slide 30
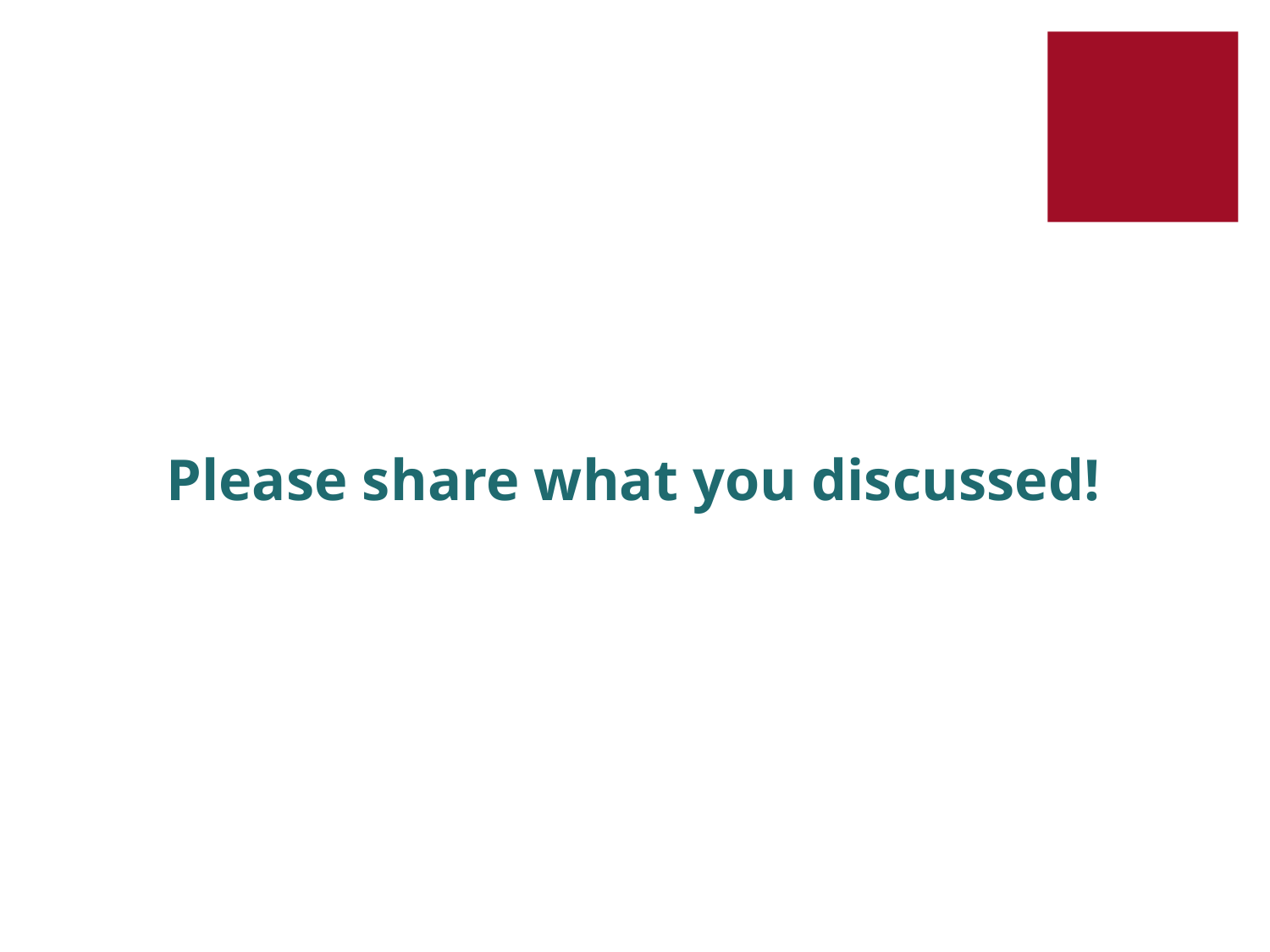

# Please share what you discussed!

## Slide 31
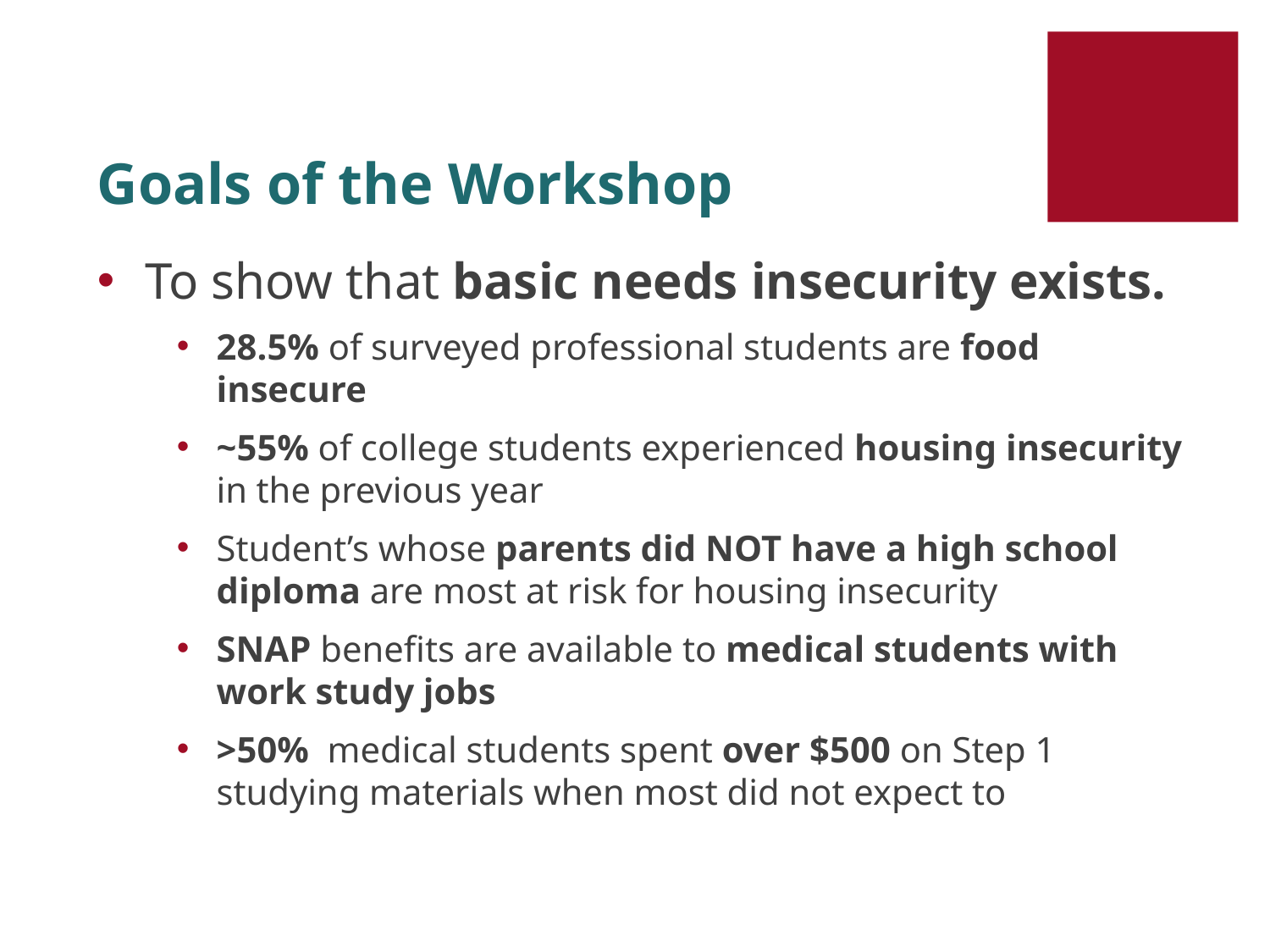

# Goals of the Workshop
To show that basic needs insecurity exists.
28.5% of surveyed professional students are food insecure
~55% of college students experienced housing insecurity in the previous year
Student’s whose parents did NOT have a high school diploma are most at risk for housing insecurity
SNAP benefits are available to medical students with work study jobs
>50% medical students spent over $500 on Step 1 studying materials when most did not expect to

## Slide 32
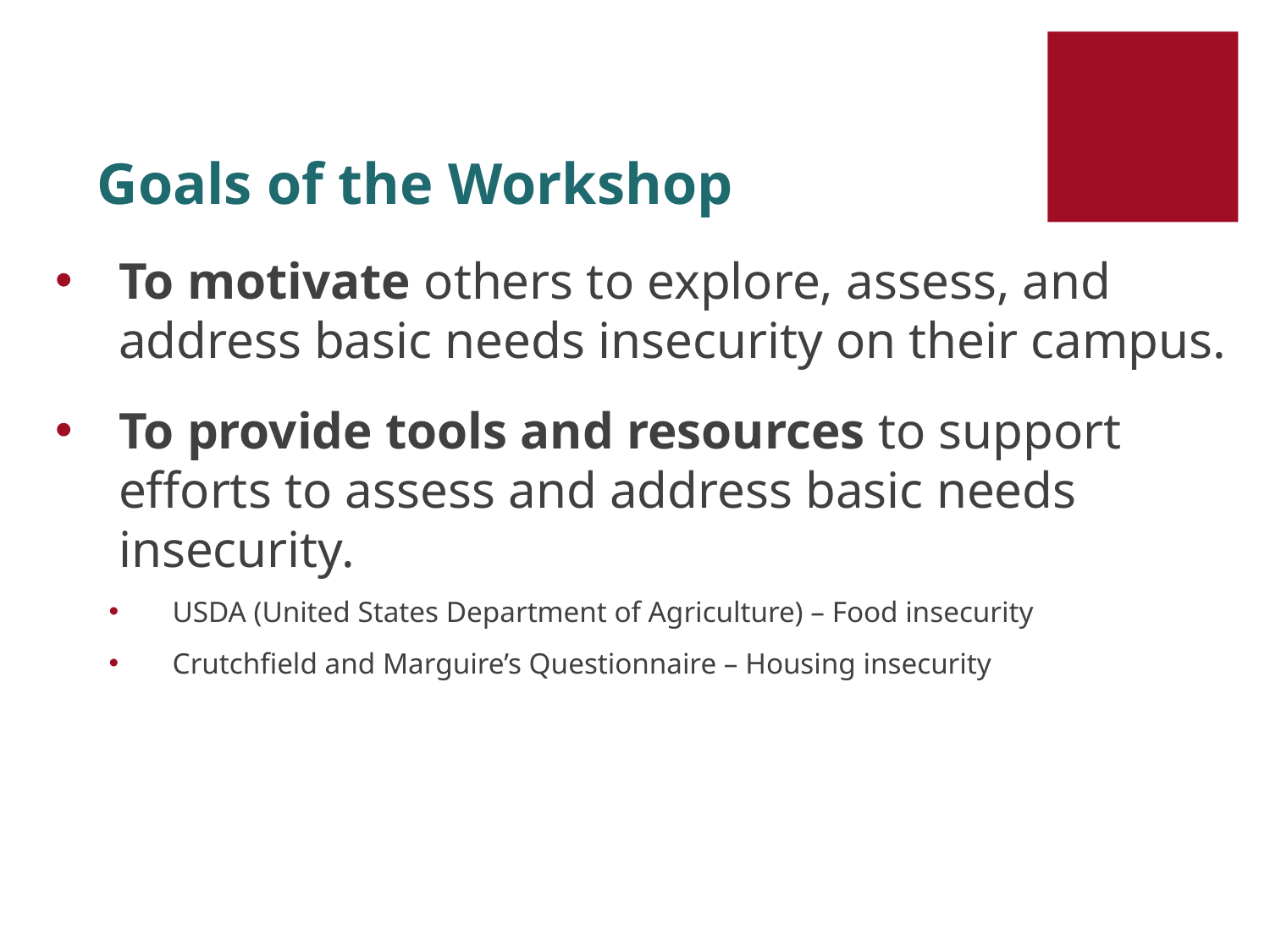

# Goals of the Workshop
To motivate others to explore, assess, and address basic needs insecurity on their campus.
To provide tools and resources to support efforts to assess and address basic needs insecurity.
USDA (United States Department of Agriculture) – Food insecurity
Crutchfield and Marguire’s Questionnaire – Housing insecurity

## Slide 33
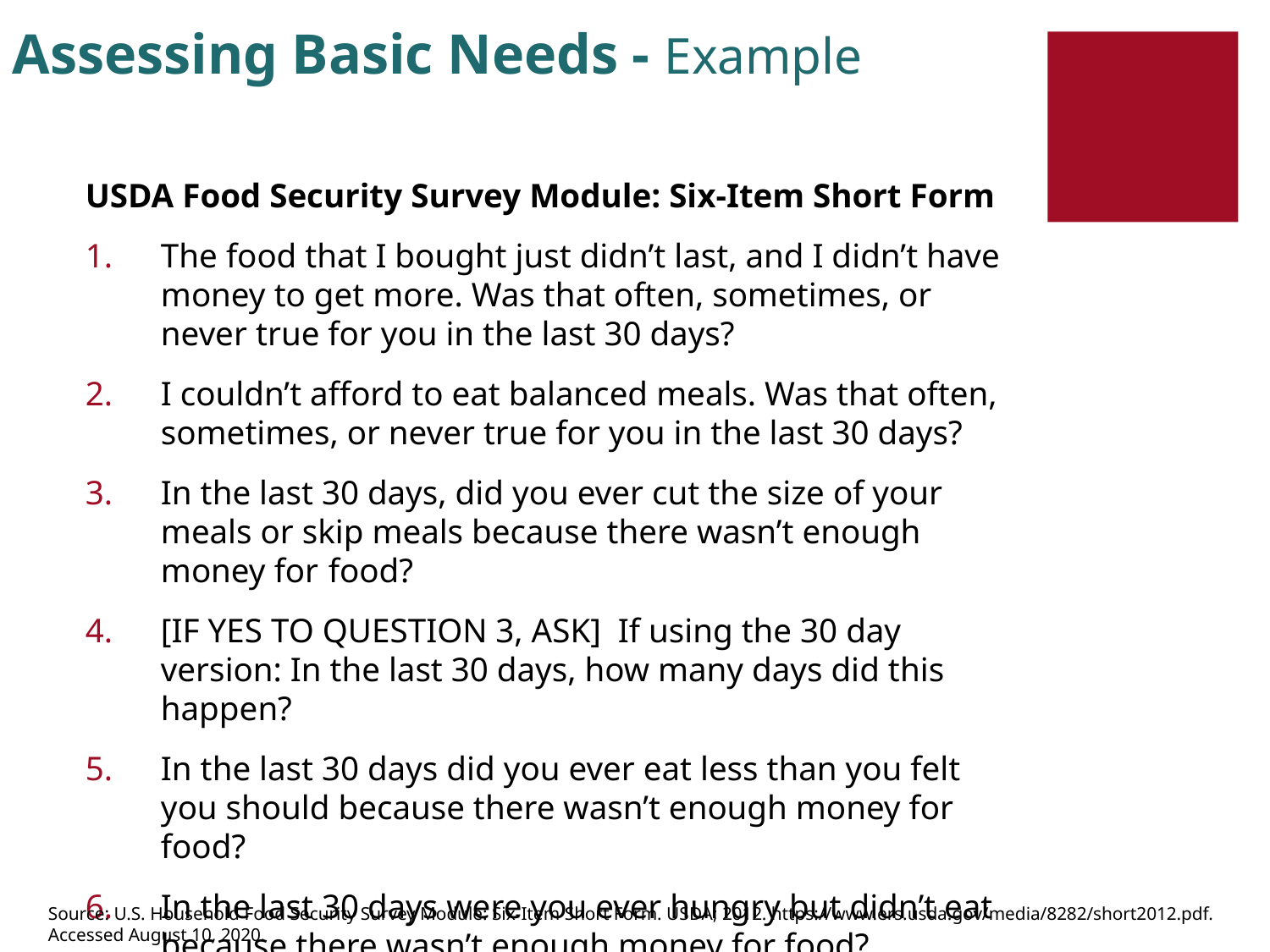

Assessing Basic Needs - Example
USDA Food Security Survey Module: Six-Item Short Form
The food that I bought just didn’t last, and I didn’t have money to get more. Was that often, sometimes, or never true for you in the last 30 days?
I couldn’t afford to eat balanced meals. Was that often, sometimes, or never true for you in the last 30 days?
In the last 30 days, did you ever cut the size of your meals or skip meals because there wasn’t enough money for 	food?
[IF YES TO QUESTION 3, ASK]  If using the 30 day version: In the last 30 days, how many days did this happen?
In the last 30 days did you ever eat less than you felt you should because there wasn’t enough money for food?
In the last 30 days were you ever hungry but didn’t eat because there wasn’t enough money for food?
Source: U.S. Household Food Security Survey Module: Six-Item Short Form. USDA; 2012. https://www.ers.usda.gov/media/8282/short2012.pdf. Accessed August 10, 2020.

## Slide 34
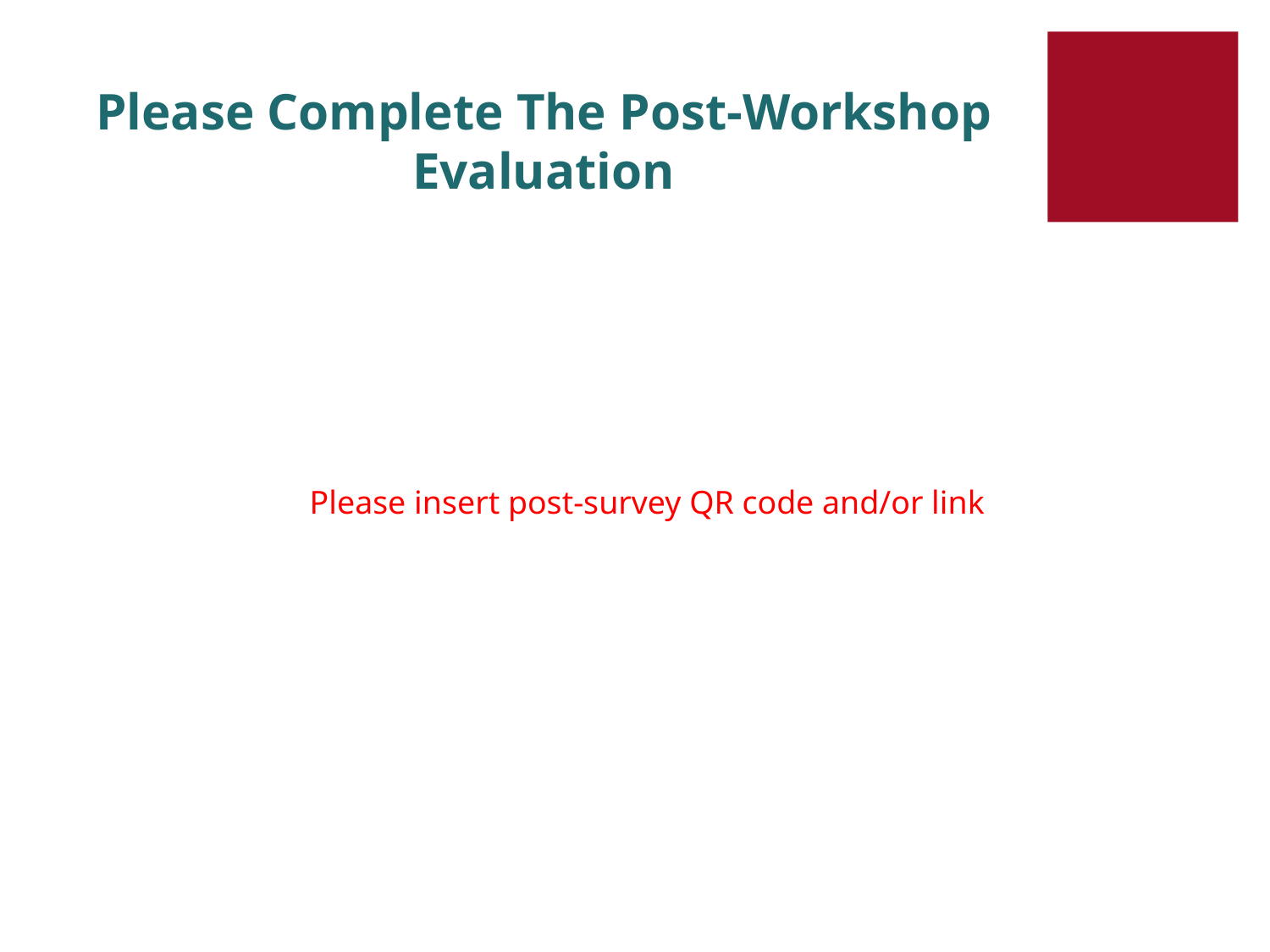

Please Complete The Post-Workshop Evaluation
Please insert post-survey QR code and/or link

## Slide 35
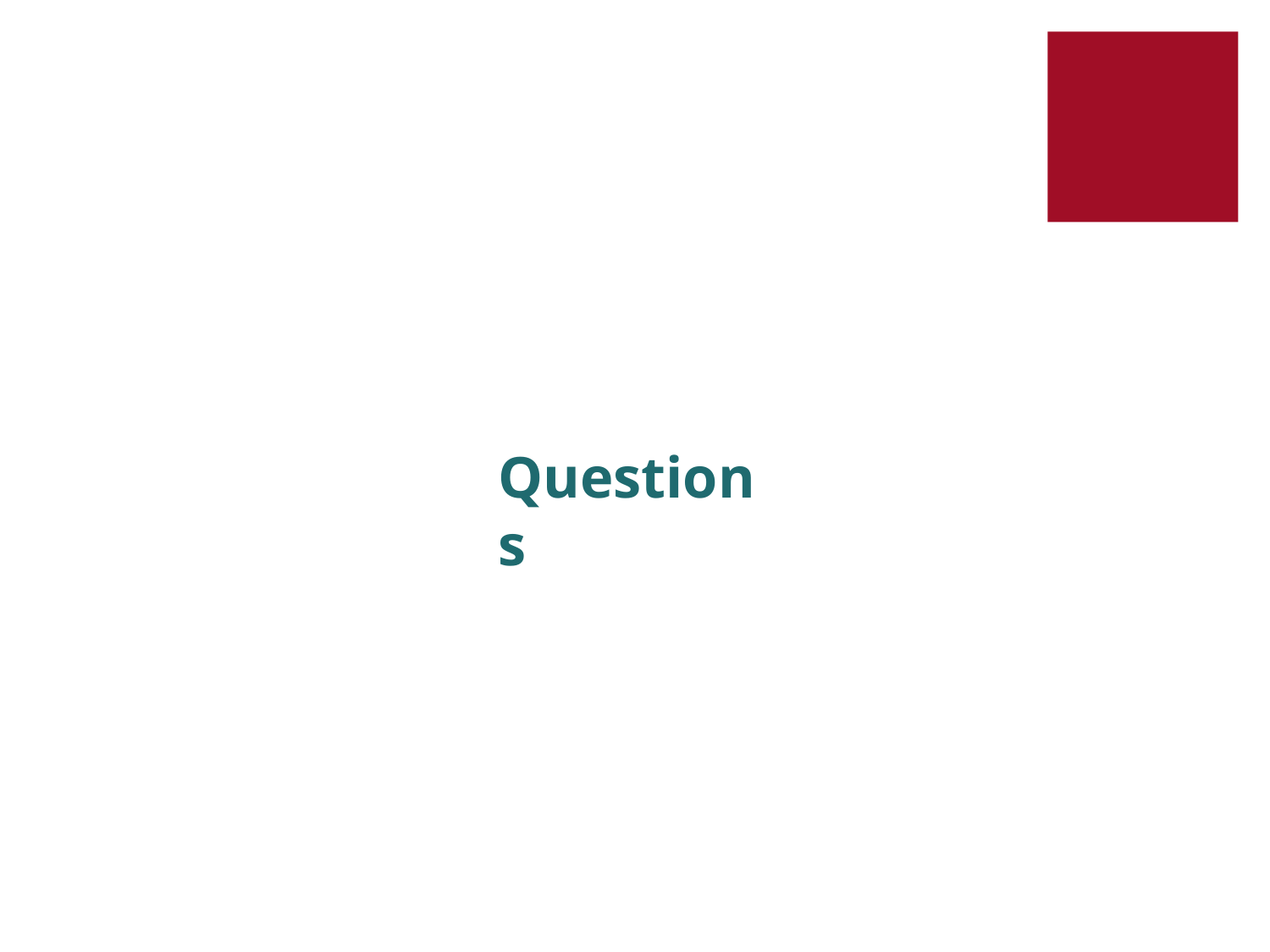

Questions

## Slide 36
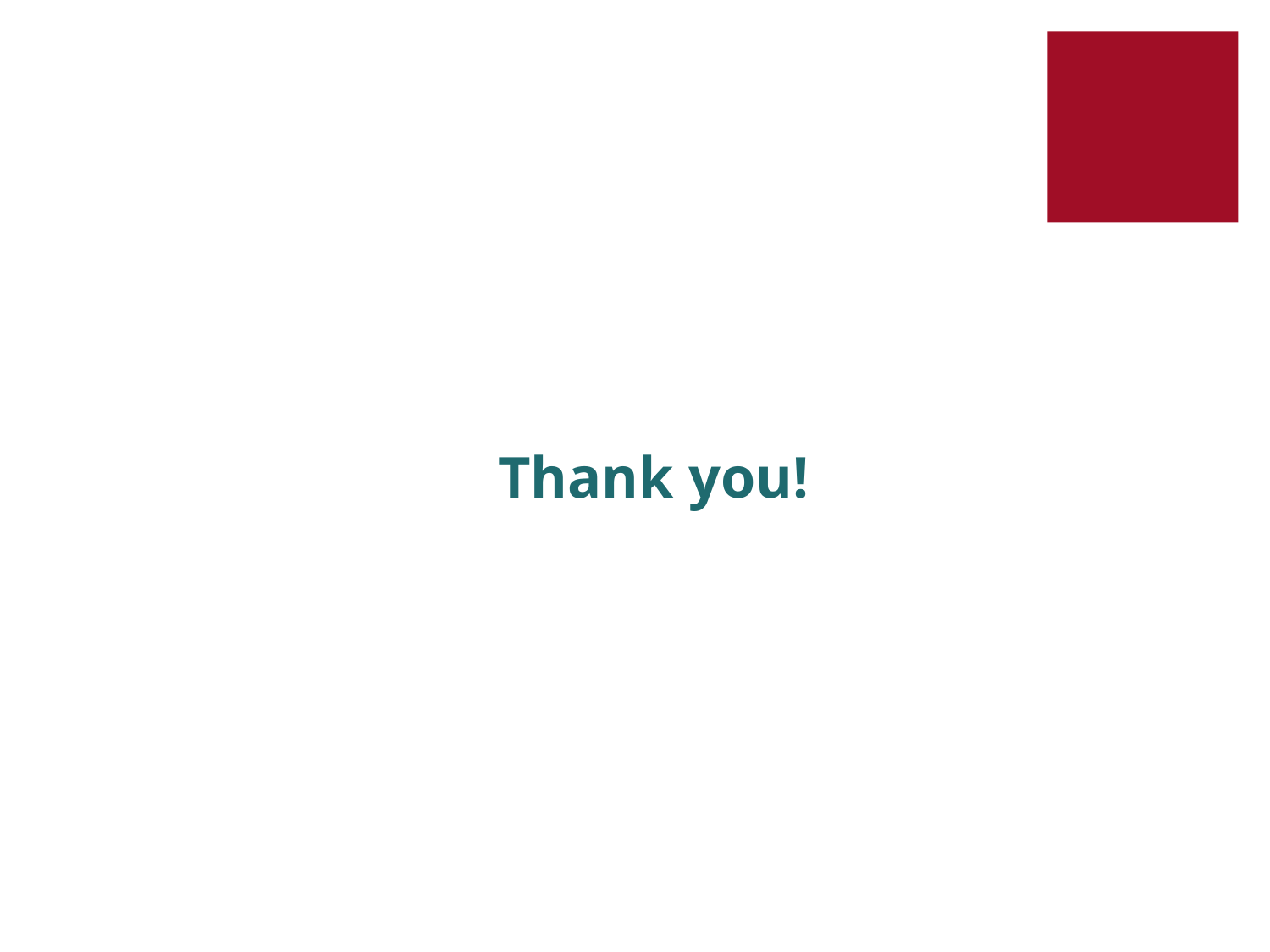

Thank you!
