# Supplementary material for: Identifying and Addressing Basic Needs Insecurity Among Medical Students: A Curriculum for Trainees, Administrators, and Faculty
Source: MedEdPORTAL. 2022 Jan 10;18:11195. doi: 10.15766/mep_2374-8265.11195 (PMC8743318; doi:10.15766/mep_2374-8265.11195)
Supplement: Supplementary file 1 — Resource Guide.docxIn-Person Facilitator Guide.docxVirtual Facilitator Guide.docxPreworkshop Survey.docxBasic Needs Presentation.pptxCase 1.docxCase 2.docxCase 3.docxPostworkshop Survey.docx [file mep_2374-8265.11195-s001.zip › F. Case 1.docx]

Case 1- Food and Academic Resource Insecurity

Gina is a second-year medical student. She grew up in a single-parent household where her mother struggled to make ends meet. Gina is the first in her family to go to college. She budgeted carefully last year to minimize the amount of debt she will be adding on to her undergraduate debt. She accepted the same amount of loan money as last year in July, expecting her cost of living to be the same since she had not moved. Discussion of purchasing her Step 1 resources began in August, and she did not account for these additional costs! She ends up spending 600 dollars on study materials from her food budget to purchase the “essential” resources her peers have bought. She has tried to pick up more hours with her work study job, but it is cutting into her time to study. She has begun rationing her food. She feels alienated from her peers because she plans most of her days around finding free food on campus. The local grocery store is overpriced, so she sticks to buying canned foods from the CVS which is walkable from her apartment.

Questions:

- What basic needs insecurities is Gina dealing with and what are the consequences of these insecurities?
- What demographic information puts Gina at risk for these insecurities?
- What community assets/resources are available for her in your community? What barriers might exist to accessing those resources and services? *(remember a Resource Guide is available as a resource)*
- What questions can we ask Gina to assess for the basic needs insecurities she may be experiencing? *(remember a Resource Guide is available as a resource)*
