# Supplementary material for: Identifying and Addressing Basic Needs Insecurity Among Medical Students: A Curriculum for Trainees, Administrators, and Faculty
Source: MedEdPORTAL. 2022 Jan 10;18:11195. doi: 10.15766/mep_2374-8265.11195 (PMC8743318; doi:10.15766/mep_2374-8265.11195)
Supplement: Supplementary file 1 — Resource Guide.docxIn-Person Facilitator Guide.docxVirtual Facilitator Guide.docxPreworkshop Survey.docxBasic Needs Presentation.pptxCase 1.docxCase 2.docxCase 3.docxPostworkshop Survey.docx [file mep_2374-8265.11195-s001.zip › G. Case 2.docx]

Case 2- Housing Insecurity

Harry is a third-year medical student. He lives with 3 other roommates to decrease his cost of living. Harry had a work study job throughout his first two years of medical school, which allowed him to break even on his expenses every month. However, he is unable to continue working 20 hours/week because of his demanding clinical rotation schedule. Harry is struggling to make rent every month, which has caused a strained relationship with his roommates. The added stress from his housing situation leaves Harry feeling exhausted and anxious, resulting in subpar performance on his clinical rotations. Harry is dealing with anxiety and depression due to his unstable living situation. He is especially concerned about the upcoming winter months because his roommates like to keep the house warm - resulting in a high monthly heating bill.

Questions

- What basic needs insecurities is Harry dealing with and what are the consequences of these insecurities?
- How could we have identified students experiencing these insecurities before the circumstances progressed to Harry’s level?
- What community or school assets/resources may be available for him on your campus?
