# Supplementary material for: Identifying and Addressing Basic Needs Insecurity Among Medical Students: A Curriculum for Trainees, Administrators, and Faculty
Source: MedEdPORTAL. 2022 Jan 10;18:11195. doi: 10.15766/mep_2374-8265.11195 (PMC8743318; doi:10.15766/mep_2374-8265.11195)
Supplement: Supplementary file 1 — Resource Guide.docxIn-Person Facilitator Guide.docxVirtual Facilitator Guide.docxPreworkshop Survey.docxBasic Needs Presentation.pptxCase 1.docxCase 2.docxCase 3.docxPostworkshop Survey.docx [file mep_2374-8265.11195-s001.zip › H. Case 3.docx]

Case 3- Transportation Insecurity

Joe is a 3rd year medical student. Joe typically walks 25 minutes to campus to avoid spending $200 each year on campus parking. He purchased a used car prior to entering medical school but uses it only when necessary. His upcoming outpatient rotation is located 30 miles from the main hospital and is not easily accessible by public transportation. It will require him to commute by car and spend $120 on parking for the month. Unfortunately, yesterday his car had trouble starting. He brought his car to the mechanic who told him the necessary repairs will cost $800. Joe had initially set aside his savings to pay for travel expenses during 4th year away rotations and residency interview season but needs his car to get to his rotation. He spends the $800 to fix his car but is now worried he will later struggle financially to do away rotations and will limit which interviews he can attend.

Questions:

- What basic needs insecurities is Joe dealing with and what are the consequences of these insecurities?
- How far in advance should students be told about extra transportation costs to allow adequate time to plan/budget?
- What community or school assets/resources may be available for him?
