# Supplementary material for: Identifying and Addressing Basic Needs Insecurity Among Medical Students: A Curriculum for Trainees, Administrators, and Faculty
Source: MedEdPORTAL. 2022 Jan 10;18:11195. doi: 10.15766/mep_2374-8265.11195 (PMC8743318; doi:10.15766/mep_2374-8265.11195)
Supplement: Supplementary file 1 — Resource Guide.docxIn-Person Facilitator Guide.docxVirtual Facilitator Guide.docxPreworkshop Survey.docxBasic Needs Presentation.pptxCase 1.docxCase 2.docxCase 3.docxPostworkshop Survey.docx [file mep_2374-8265.11195-s001.zip › I. Postworkshop Survey.docx]

Post-Workshop Survey

Q1 Which is a basic need of medical students?

- **Academic resources to ensure they succeed.**
- A car so they can get to clinical rotations.
- A mentor they trust for career advice.
- A free gym membership to maintain their physical and mental health.

Q2 What percent of professional students were found to be food insecure in the 2018 survey?

- 5%
- **28.5%**
- 45%
- 75.9%

Q3 What percent of college students experience housing insecurity?

- 5%
- 15%
- **55%**
- 70%

Q4 Which student characteristic is most predictive of their likelihood for experiencing housing insecurity?

- Race/Ethnicity - American Indian/Alaskan Native students
- **Parental Education - Students whose parents do not have a high school diploma**
- Older Students - Students aged 26-30
- Sexual Orientation - Gay and lesbian students

Q5 Which medical student is available for Supplemental Nutrition Assistance Program (SNAP) benefits?

- No medical student, it is only for undergraduate students
- **A medical student with work study job**
- A medical student whose parents make <$100,000 annually
- A medical student who is in the top 15% of their class

Q6 To what extent do you agree that the workshop learning objectives were met?

|  | Strongly agree | Somewhat agree | Neither agree nor disagree | Somewhat disagree | Strongly disagree |
| --- | --- | --- | --- | --- | --- |
| You understand 'Basic Needs Insecurity' (BNI). |  |  |  |  |  |
| You understand 'Food Insecurity'. |  |  |  |  |  |
| You understand 'Housing Insecurity'. |  |  |  |  |  |
| You understand 'Academic Resource Insecurity'. |  |  |  |  |  |
| You understand the extent to which BNI exists among students in higher education. |  |  |  |  |  |
| You can identify a tool to assess for BNI. |  |  |  |  |  |
| You can outline strategies to support students if they are experiencing BNI. |  |  |  |  |  |

Q7 What did you like about this workshop?

________________________________________________________________

________________________________________________________________

________________________________________________________________

________________________________________________________________

________________________________________________________________

Q8 What suggestions do you have to improve this workshop?

________________________________________________________________

________________________________________________________________

________________________________________________________________

________________________________________________________________

________________________________________________________________
