# Supplementary material for: Identifying and Addressing Basic Needs Insecurity Among Medical Students: A Curriculum for Trainees, Administrators, and Faculty
Source: MedEdPORTAL. 2022 Jan 10;18:11195. doi: 10.15766/mep_2374-8265.11195 (PMC8743318; doi:10.15766/mep_2374-8265.11195)
Supplement: Supplementary file 1 — Resource Guide.docxIn-Person Facilitator Guide.docxVirtual Facilitator Guide.docxPreworkshop Survey.docxBasic Needs Presentation.pptxCase 1.docxCase 2.docxCase 3.docxPostworkshop Survey.docx [file mep_2374-8265.11195-s001.zip › Educational_Summary_Report_11195.pdf]

MedEdPORTAL® The AAMC Journal of Teaching and Learning Resources

Original Publication

Open Access

Identifying and Addressing Basic Needs Insecurity Among Medical Students: A Curriculum for Trainees, Administrators, and Faculty

Michele Cerasani*, Enxhi Rrapi, Tanuj Sharma, Angelina Ferritto, Mytien Nguyen, MS, Ryan Barrette, Hyacinth Mason, MPH, PhD, CHES

*Corresponding author: Michele.Cerasani@gmail.com

Abstract

Introduction: Data from a 2018 United States Government Accountability Office report show that basic needs insecurity undermines the postsecondary educational experiences of many students. In recent years, basic needs insecurity among undergraduate students has gained attention in the literature, but published data regarding medical trainees are extremely limited. **Methods:** A 60-minute interactive workshop consisting of a PowerPoint presentation and case discussions was created. Our aim was to increase awareness and understanding of basic needs insecurity among medical students. The workshop included a basic needs survey and national and local resource guides. The workshop was evaluated through pre- and postworkshop questionnaires. **Results:** There were a total of 61 participants with diverse identities, including premedical and medical students, faculty, staff, and administrators. A comparison of pre- and postworkshop data showed increases in all knowledge-based questions, two of which were statistically significant. Most learners somewhat or strongly agreed the learning objectives were met. Participants positively commented on the interactive and collaborative nature of the workshop, the perspective the case discussions offered, and the tangible resources provided to them. **Discussion:** This single session serves as a starting point to bring awareness that basic needs insecurity exists among medical trainees. It is a step toward a cross-departmental approach to assess the scope of the problem and find solutions to address it. Through the widespread implementation of this session, we hope participants can enact sustainable institutional changes that will support the basic needs of students.

Keywords
Basic Needs Insecurity, Food Insecurity, Housing Insecurity, Transportation Insecurity, Academic Resource Insecurity, Case-Based Learning, Online Learning, Distance Learning, Diversity, Inclusion, Health Equity

Educational Objectives

By the end of this activity, learners will be able to:

1. Define basic needs insecurity of medical trainees.
2. Assess for basic needs insecurity.
3. Describe the prevalence of basic needs insecurity among students in higher education.
4. List at least one effective practice or policy that can be implemented at medical schools to address basic needs insecurity.

Citation:
Cerasani M, Rrapi E, Sharma T, et al. Identifying and addressing basic needs insecurity among medical students: a curriculum for trainees, administrators, and faculty. *MedEdPORTAL*. 2022;18:11195.
https://doi.org/10.15766/mep_2374-8265.11195

Introduction

Data from a 2018 United States Government Accountability Office report show that basic needs insecurity undermines the postsecondary educational experiences of many students.[1-3] Traditionally, basic needs are considered in terms of food insecurity or housing insecurity. Food insecurity refers to the uncertainty about or limited availability of nutritionally adequate food.[4] Housing insecurity is defined as the inability to pay rent or utilities, moving often, and not having a permanent safe place to live.[2,3] This is an important topic, albeit understudied in the current literature, particularly among medical students. When discussing basic needs in relation to medical students, it is necessary to consider their additional needs. Two added basic needs for the success of medical trainees include academic tools for success and transportation to clinical sites. Academic tools for success insecurity refers to the difficulty or inability to afford resources required or recommended to be properly prepared for academics. Transportation insecurity refers to the inability to regularly move from place to place in a safe and timely

Copyright © 2022 Cerasani et al. This is an open-access publication distributed under the terms of the Creative Commons Attribution license.

manner due to a lack of resources.[5] Transportation is considered essential since medical students are often required to travel to clinical rotations.

Published data on the basic needs insecurity of medical trainees are limited. We hypothesized that medical students are struggling to meet their basic needs, resembling the trends found among college students. Based on a 2018 survey of nearly 86,000 college students from across the US, it was found that basic needs insecurity disproportionately affected various student populations. The rates of basic needs insecurity were higher for students who identified as Black or African American, American Indian or Alaska Native, Pacific Islander or Native Hawaiian, and Hispanic or Latinx, compared to those who identified as Middle Eastern or North African or Arab, Southeast Asian, and White or Caucasian.[4] Other student characteristics associated with higher rates of basic needs insecurity included those who were employed, served in the military, attended 2-year rather than 4-year institutions, or were over the age of 26.[4] In addition, college students who identified as transgender were more likely to experience basic needs insecurity than students who identified as cisgender.[4] Varying levels of support may be available depending on institution, but even when support exists, there is evidence that some students avoid utilizing available resources due to the associated social stigma.[5]

Among the small amount of data available in the literature, researchers in 2018 surveyed over 2,000 health professional trainees, which included nursing, dental, and medical students, and found that the prevalence of food insecurity was 28%.[6] Among students who were food insecure, one in five reported that it impacted their academic performance.[6] Although published data on the basic needs insecurity of medical trainees specifically are extremely limited, we hypothesized that increased attention, research, and solutions regarding basic needs insecurity are needed, as it is silently undermining the education and development of future physicians.

To shed light on this issue, we created a curriculum in the form of a 60-minute, single-session workshop. Kern's six-step model for curriculum development was used to design our workshop.[7] In step one, a problem identification and general needs assessment was completed. This included a review of the literature, which revealed no prior *MedEdPORTAL* publications on the topic of basic needs among medical trainees. In step two, we developed a targeted needs assessment using both published resources and newly developed questionnaires to glean the prevalence of basic needs insecurity existing among medical trainees at our institution. In step three, we developed our learning objectives based on the literature review and results from the needs assessment. In step four, educational strategies included the creation of a PowerPoint presentation and case discussions. In step five, we conducted the workshop four times, online and in person, making iterative changes as needed to its content and timing. In the last step, evaluation and feedback through an online survey were developed to determine the extent to which our learning objectives were achieved. The protocol and evaluation tools were approved by the Albany Medical College Institutional Review Board (IRB).

The purpose of this workshop was to motivate the exploration of, assessment of, and response to the basic needs insecurity of medical learners by engaging medical school administrators, faculty, staff, and medical and premedical students. Cross-departmental approaches are needed to understand the scope and scale of these issues, destigmatize them, and strategize ways to address them.

Methods

This 60-minute workshop aimed to explore, assess, and address basic needs insecurity among medical trainees through a presentation and case study approach. The workshop was developed by faculty and students with diverse personal and professional backgrounds. The senior author used her experience as assistant dean for student support and inclusion to compile concerns raised by medical students and to create a workshop to investigate the needs of medical trainees. The students—who came from first-generation, low-income, and/or minority backgrounds—were able to use their lived experiences to enhance the content. This workshop was intended to be piloted with medical education professionals at the 2020 Northeast Group on Educational Affairs AAMC Conference, but the conference was cancelled due to the COVID-19 pandemic.

Two iterations of the workshop were developed, one in which the curriculum was conducted virtually to allow learners from multiple institutions to participate. The other version was conducted in person for learners at a single institution. Learners for both versions included administrators/staff, academic faculty, medical students, and premedical students. The curriculum served the purpose of the ACGME and faculty education concerning diversity, equity, and inclusion and was implemented for a variety of learners invested in supporting medical students. IRB approval was obtained to run the workshop at Albany Medical College and to survey participants for publishing purposes.

A basic needs survey was sent to all medical students at our institution prior to the implementation of this curriculum

Copyright © 2022 Cerasani et al. This is an open-access publication distributed under the terms of the Creative Commons Attribution license.

(Appendix A). An email was sent out to all medical students that included a link to submit the survey anonymously. Understanding the rates of basic needs insecurity prior to offering the in-person session at a single institution was deemed important to quantify the problem of the student population that would be discussed during the workshop.

Medical students, premedical students, residents, faculty, and administrators were recruited via email to participate in the workshop through the National First-Generation and Low-Income Students in Medicine (FGLIMed) and Student National Medical Association (SNMA) student groups. We implemented and evaluated the workshop four times—in person (once) and then virtually (thrice)—with participants coming from over 10 different academic institutions. All the virtual workshops were run through nonaffiliated sites using Zoom. Each workshop was led by two facilitators and one technical moderator (if virtual) using the facilitator guide (Appendix B or C). Facilitators included premedical and medical students. Facilitators were not required to have preexisting knowledge of basic needs insecurity since the facilitator guide provided sufficient background and guidance to successfully lead the workshop. Similarly, no previous knowledge of basic needs insecurity was needed by participants.

Prior to the start of the workshop, the resource guide was shared with learners, and they were encouraged to reference it during the workshop. All participants were asked to take the preworkshop survey (Appendix D) once they logged onto Zoom or entered the in-person conference room. The presurvey assessed participants' knowledge of basic needs insecurity before the workshop by asking multiple-choice questions. The first facilitator began by leading the PowerPoint presentation (Appendix E) during which learners brainstormed the basic needs challenges facing medical trainees. The facilitator defined basic needs and related terminology, presented statistics, and shared examples of how insecurities might be alleviated. Then, as one large group, participants discussed a case of a medical student (Appendix F) with basic needs insecurities. Afterward, learners were broken into two groups no larger than 10 people each. One facilitator led each small group, where they guided learners through different cases (Appendix G or H) of a medical student with at least one basic need not being met. Learners were encouraged to integrate information from the presentation and resource guide, as well as personal experiences, to answer the facilitators' questions related to their respective case. Next, everyone reconvened to discuss their cases as a large group. A spokesperson from each small group shared the major takeaways of their case. One facilitator then closed the session by reviewing the main conclusions from the workshop. Before leaving, participants completed the postworkshop evaluation survey (Appendix I), which asked the same multiple-choice questions as the presurvey to assess the effectiveness of the workshop.

All workshops were evaluated using pre- and postsurveys completed by participants that were then reviewed by our team. The workshop and surveys were refined in an iterative manner with each subsequent run using participant feedback to improve the workshop's effectiveness. The curriculum was interactive and engaged learners through the large-group PowerPoint presentation, small-group case discussions, and numerous opportunities for participants to share their perspectives.

Results

Sixty-one participants attended the workshop, of whom 53 (87%) and 45 (74%) completed the pre- and postworkshop questionnaires, respectively. Participants were in New York, Massachusetts, Georgia, and New Hampshire. Data from these 53 participants were included in the statistical analysis. Demographic data (Table 1) were available for 41 of 53 participants who completed the preworkshop questionnaire; 49% of respondents identified as first-generation college graduates, 51% were Pell Grant recipients, 73% identified as female, and 39% identified as underrepresented in medicine (Black/African American and Latinx).

Results from the knowledge-based questions on the pre- and postsurveys demonstrated a trend towards enhanced

Table 1. Participant Characteristics

Demographic	No. (%)
Total	41 (100)
Position	
Undergraduate student	4 (10)
Medical student	29 (71)
Administrator	4 (10)
Other	4 (10)
Race/ethnicity	
Asian	9 (22)
Black/African American	12 (29)
Latinx/Hispanic	4 (10)
White	9 (22)
Other	2 (5)
Parental education	
Continuing-generation college graduate	21 (51)
First-generation college graduate	20 (49)
Sex	
Male	11 (27)
Female	30 (73)
Pell Grant recipient	
No	20 (49)
Yes	21 (51)

Copyright © 2022 Cerasani et al. This is an open-access publication distributed under the terms of the Creative Commons Attribution license.

understanding of basic needs insecurity, with participants showing statistically significant improvement ($p < .01$) on the average score of some questions from pre- to postworkshop (Table 2). An assessment regarding the effectiveness of the workshop in meeting the learning objectives is summarized in the Figure. Overall, most participants strongly agreed that the workshop objectives, which included understanding basic needs, food, housing, and academic needs insecurities, were met. Although most participants somewhat agreed or strongly agreed that they understood the extent that basic needs insecurity existed on campus, could identify tools for basic needs insecurity, and could outline strategies to support students with basic needs insecurity, a few responded neutrally, and two somewhat disagreed that the workshop met a specific objective. The two responses that indicated a participant somewhat disagreed occurred during the first iteration of the workshop.

Discussion

Our curriculum was designed to increase awareness of basic needs insecurity existing among medical trainees and to encourage medical school administrators, faculty, staff, and students to identify and develop institutional responses. A strength of this curriculum is the common outcome of participants learning to define the terminology of basic needs insecurity as well as identify possible interventions to improve equity by eliminating food, housing, transportation, or academic resource insecurity among medical students. Overall, participants largely agreed that the workshop was effective in meeting these learning objectives and showed improvement in their knowledge of the topic, as indicated by the pre- and postworkshop survey results. Respondents commended the small-group case discussions and the tangible tools the workshop provided and most particularly appreciated discussing the first case as a large group and subsequently moving into smaller groups to discuss remaining cases. Others commented that the workshop nicely introduced ideas and concepts and then allowed them the chance to self-reflect and share personal thoughts about the case studies.

The curriculum was presented successfully in both in-person and virtual formats. The facilitator guide documents (Appendices B and C) enabled volunteers with a wide range of experience to successfully facilitate these sessions in both formats. Distribution of the resource guide (Appendix A) prior to the session ensured all participants shared a baseline fund of knowledge and offered a resource to refer to during the workshop if clarification of a

Table 2. Workshop Survey Responses

	Percent Correct		
Question	Preworkshop	Postworkshop	*p*
Which is a basic need of medical students?	56	68	.280
What percent of professional students are food insecure?	32	71	<.001
What percent of college students experience housing insecurity?	34	76	<.001
Which student characteristic is most predictive of their likelihood for experiencing housing insecurity?	53	70	.081
Which medical student is available for SNAP benefits?	73	88	.104

Abbreviation: SNAP, Supplemental Nutrition Assistance Program.

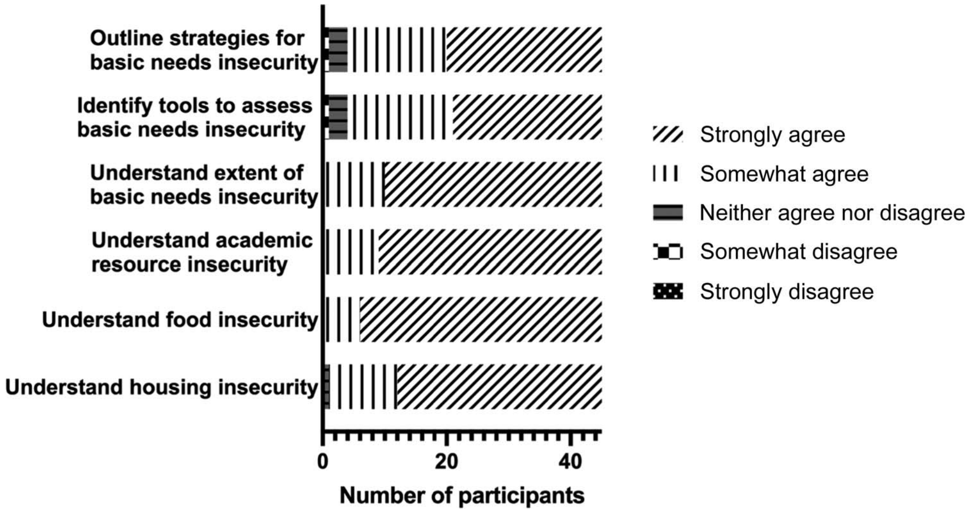

Figure. Participant perspectives on the achievement of workshop learning objectives.

Copyright © 2022 Cerasani et al. This is an open-access publication distributed under the terms of the Creative Commons Attribution license.

topic presented in the session was needed. Importantly, the diversity of learners represented allowed for rich, interdisciplinary discussions. Additionally, the cases (Appendices F-H) were designed with multiple layers of complexity, which made them suitable for participants across a range of expertise.

Limitations of this study included the small sample size ($N = 61$). Furthermore, of the 41 participants who filled out their demographics, women (73%), White (22%) and Black/African American (29%) participants were disproportionately represented compared to national data on US medical school enrollment (52%, 48%, and 8%, respectively).[8] The overrepresentation of women (73%) and first-generation college graduates (48%) in our sample reflected the membership of the student organizations from which many participants were recruited, particularly FGLIMed and SNMA. It is possible that first-generation college graduates and other marginalized students may have had higher awareness of basic needs insecurity compared to their peers and may have introduced self-selection bias. Although this might be the case, our experience presenting this session indicates that it has motivated diverse groups of students to learn more about the level of basic needs in their student community and take action to end basic needs insecurity. Large-scale investigations are required to further characterize the national awareness of medical trainee basic needs insecurity. Surveying students for basic needs insecurity and implementation of this curriculum at additional institutions would help generalize the results to medical trainees nationally. Additionally, as seen in Table 2, while the postsurvey responses were higher, only two of our five questions showed a statistically significant improvement. This outcome may be the result of the iterative changes made to improve the workshop with each subsequent run based on participant feedback. After our first pilot session, it was noticed that several questions in the pre- and postworkshop questionnaires were inadequately addressed in the presentation; therefore, we modified the PowerPoint accordingly.

Although our learners were primarily premedical and medical students, faculty play key roles in the professional development of medical students including both formal advising roles and informal mentoring roles. Through our session, we hope to empower participants (administrators, medical school leadership, faculty members, and medical students) to educate and advocate for their faculty and administration to act on an institutional level, as well as in roles in national organizations and committees. Unfortunately, the onus of this issue has fallen on medical students to provide peer support as well as advocacy for meaningful attention to be brought to this issue. Ultimately, the goal of our session was to stress the need for faculty and administrators to engage in this topic to create long-lasting instructional changes. Due to the high turnover of student leaders, institutional support is required for long-term sustainability of initiatives such as food pantries, community gardens, and financial relief efforts. Furthermore, administrators are also able to procure financial resources on a larger scale to address this issue. We believe this module is appropriate for professional development sessions for faculty and aids in our quest to ensure all in health professions education learn to recognize and effectively respond to student resource insecurity. Offering this session during protected academic time, such as during departmental conferences, or offering graduate medical education credit for the session could help attract more participation from faculty/administrators in the future.

Our curriculum offers two formats for the workshop, although differences between virtual and in-person sessions have not been investigated. Virtual experience may hinder some participants from active participation. However, the virtual format allows interaction between institutions from different regions, which likely enriches the content of discussion. While the decision to use a virtual format was largely driven by the logistical needs of the COVID-19 pandemic, future iterations of the workshop may still benefit from a virtual or hybrid model to facilitate interinstitutional dialogue.

Future directions for this curriculum include implementation at additional institutions as well as expanding the diversity of learners within institutions. Furthermore, the workshop can be used as a framework for institutional leaders to develop their own cases to discuss issues of resource access unique to their program. In addition, a future subgroup analysis of subsequent surveys may elucidate differences in basic needs insecurity awareness between groups, such as students and faculty. If present, such differences may indicate the need for targeted interventions to improve awareness of medical trainee needs. Overall, the single-session format presented here serves as a starting point to bring awareness to basic needs insecurity among medical trainees as well as to assess the scope of the problem and begin to find solutions.

Appendices

A. Resource Guide.docx

B. In-Person Facilitator Guide.docx

Copyright © 2022 Cerasani et al. This is an open-access publication distributed under the terms of the Creative Commons Attribution license.

C. Virtual Facilitator Guide.docx
D. Preworkshop Survey.docx
E. Basic Needs Presentation.pptx
F. Case 1.docx
G. Case 2.docx
H. Case 3.docx
I. Postworkshop Survey.docx

All appendices are peer reviewed as integral parts of the Original Publication.

Michele Cerasani: Third-Year Medical Student, Albany Medical College

Enxhi Rrapi: Second-Year Medical Student, Albany Medical College

Tanuj Sharma: First-Year Doctoral Candidate, University at Albany

Angelina Ferritto: Undergraduate Student, Emory University

Mytien Nguyen, MS: Fifth-Year Medical Student and Doctor of Philosophy Candidate, Yale University School of Medicine

Ryan Barrette: Fourth-Year Medical Student, University of Massachusetts Medical School

Hyacinth Mason, MPH, PhD, CHES: Assistant Dean, Student Support and Inclusion, Albany Medical College; Associate Professor, Department of Medical Education, Albany Medical College

Acknowledgments
We thank the National First-Generation and Low-Income Medical Student Association (FGLIMed) and Albany Medical College's FGLIMed chapter, Dr. Branden Eggan, Michelle Williams, and Nicholas Brutus for their contributions.

Disclosures
None to report.

Funding/Support
None to report.

Ethical Approval
The Albany Medical College Committee on Research Involving Human Subjects Institutional Review Board approved this project.

References

1. *Food Insecurity: Better Information Could Help Eligible College Students Access Federal Food Assistance Benefits.* United States Government Accountability Office; 2018. GAO-19-95. Accessed December 16, 2020. https://www.gao.gov/assets/gao-19-95.pdf
2. Elzein A, Shelnutt K, Colby S, et al. The prevalence of food insecurity and its association with health and academic outcomes among college freshmen. *Adv Nutr*. 2017;8(1):4. https://doi.org/10.1093/advances/8.1.4
3. Broton KM, Goldrick-Rab S. Going without: an exploration of food and housing insecurity among undergraduates. *Educ Res.* 2018; 47(2):121-133. https://doi.org/10.3102/0013189X17741303
4. Goldrick-Rab S, Baker-Smith C, Coca V, Looker E, Williams T. *College and University Basic Needs Insecurity: A National #RealCollege Survey Report*. The Hope Center; 2019. Accessed January 31, 2021. https://hope4college.com/wp-content/uploads/2019/04/HOPE_realcollege_National_report_digital.pdf
5. Gould-Werth A, Griffin J, Murphy AK. Developing a new measure of transportation insecurity: an exploratory factor analysis. *Surv Pract*. 2018;11(2):1-34. https://doi.org/10.29115/SP-2018-0024
6. DeMunter J, Rdesinski R, Vintro A, Carney PA. Food insecurity among students in six health professions' training programs. *J Stud Aff Res Pract*. 2021;58(4):372-387. https://doi.org/10.1080/19496591.2020.1796690
7. Thomas PA, Kern DE, Hughes MT, Chen BY, eds. *Curriculum Development for Medical Education: A Six-Step Approach.* 3rd ed. Johns Hopkins University Press; 2016.
8. *2020 FACTS: Enrollment, Graduates, and MD-PhD Data: Table B-3: Total US Medical School Enrollment by Race/Ethnicity (Alone) and Sex, 2016-2017 Through 2020-2021*. Association of American Medical Colleges; 2020. Accessed January 28, 2021. https://www.aamc.org/media/6116/download

Received: March 25, 2021
Accepted: August 17, 2021
Published: January 10, 2022

Copyright © 2022 Cerasani et al. This is an open-access publication distributed under the terms of the Creative Commons Attribution license.
